# Persistence of atoms in molecules: there is room beyond electron densities

**DOI:** 10.1107/S2052252524000915

**Published:** 2024-02-20

**Authors:** María Menéndez-Herrero, Ángel Martín Pendás

**Affiliations:** a Dpto. Química Física y Analítica, Universidad de Oviedo, 33006 Oviedo, Spain; Tsinghua University, China

**Keywords:** computational modeling, density functional theory, molecular simulations, energy minimization, electron densities, Born maxima

## Abstract

Evidence that the electronic structure of atoms persists in molecules to a much greater extent than has been usually admitted is presented. This is achieved by resorting to *N*-electron real-space descriptors instead of one- or at most two-particle projections like the electron or exchange-correlation densities. Here, the 3*N*-dimensional maxima of the square of the wavefunction, the so-called Born maxima, are used. Since this technique is relatively unknown to the crystallographic community, a case-based approach is taken, revisiting first the Born maxima of atoms in their ground state and then some of their excited states. It is shown how they survive in molecules and that, beyond any doubt, the distribution of electrons around an atom in a molecule can be recognized as that of its isolated, in many cases excited, counterpart, relating this fact with the concept of energetic promotion. Several other cases that exemplify the applicability of the technique to solve chemical bonding conflicts and to introduce predictability in real-space analyses are also examined.

## Abstract 2

The 3*N*-dimensional maxima of the square of the wavefunction, the so-called Born maxima, show beyond doubt that the electronic structure of atoms persists in molecules, either in their original ground state or some low-lying excited state. The electron density is only a low-dimensional projection of this much richer landscape.

## 1. Introduction

At the heart of chemistry lies the fundamental pursuit of finding patterns and underlying principles that govern the behavior of matter. This quest for order and organization is an essential aspect of the scientific endeavor. In the early 19th century, the field of chemistry underwent a transformative period as chemists sought to make sense of the numerous elements and their properties. By 1808, John Dalton (Dalton, 1808 ▸) had made a significant breakthrough by organizing the known elements based on atomic weights. This marked a crucial step in understanding the composition of matter and laid the foundation for modern atomic theory. Atomic weights remained instrumental for about a century, leading with the help of a large number of great scientists to Mendeleev’s periodic table of the elements in 1869 (Scerri, 2007 ▸). About 30 years later, the discovery of X-rays by Röntgen in 1895 (Röntgen *et al.*, 2015 ▸) and of the electron of Stoney by J. J. Thomson in 1897 (Thomson, 1897 ▸) shaked Dalton’s indivisible atom and paved the way to understanding its inner structure.

During this turbulent time of change, X-rays played a fundamental role. On the one hand, Henry Moseley found in 1913 a remarkable regularity between the frequency of the *K*α emission lines of a range of elements and an integer that coincided with their ordinal numbers in the periodic table. On the other hand, Sommerfeld suggested that X-rays possessed a wavelength of ∼1 Å, and the idea that crystals could be used as diffraction gratings for X-rays arose in a conversation between Ewald and von Laue in 1912 that culminated in a paper later that year that showed clear diffraction peaks in a copper sulfate crystal (Eckert, 2012 ▸). During 1912 and 1913, the Braggs solved the structures of a number of simple crystals, including those of table salt and diamond (Bragg, 1913 ▸), and soon it became possible to obtain reliable atomic positions for more complex unit cells. In 1916, Gilbert N. Lewis wrote his seminal paper ‘The atom and the molecule’ (Lewis, 1916 ▸) laying the foundations of modern valence theory, proposed the cubical atom and even attributed the high stability of systems like NaCl to the existence of filled shells. Remarkably, in 1922 the younger Bragg re-examined previously published data in rock salt to propose an experimental electron density that was further partitioned into those of isolated Na^+^ and Cl^−^ ions. The atomic shell structure came out vividly in that work (Bragg *et al.*, 1922 ▸), which found seven electrons on a shell of radius 0.29 Å and three on a shell of radius 0.75 Å for Na^+^. Given the technical means of the time, and taking into account that modern wave mechanics was still to emerge, these achievements are truly astonishing. These insights were obtained years before the Schrödinger equation was proposed, without a guiding Pauli principle that would forbid three electrons in the cores, and when only Bohr–Sommerfeld quantization conditions were used.

Following the development of quantum mechanics, electron shells and subshells in atoms were successfully related to the quantization of orbital angular momentum, and Heitler & London (1927 ▸) showed for the first time that the new paradigm was also capable of dealing with chemical bonds. Although in Heitler & London’s original method the atoms did not completely lose their individuality – the molecular wavefunction was built from those of the atoms – and this is to some extent inherited in modern valence bond theory, soon Mulliken and Hund introduced an alternative scheme (Mulliken, 1930 ▸), molecular orbital theory, in which the basic unit moves from the atom to the electron, which fills one-particle molecular states in much the same way as occurs in the case of atoms. It soon became clear that as two quantum mechanical objects were allowed to interact, they became inextricably entangled with each other. In this way, atoms dissolved in the sea of quantum mechanics, as did many of the cherished concepts that, like the chemical bond, had been heuristically introduced by the chemists. Citing Coulson (1955 ▸): ‘Sometimes it seems to me that a bond between two atoms has become so real, so tangible, so friendly that I can almost see it. And then I awake with a little shock: for a chemical bond is not a real thing: it does not exist: no-one has ever seen it, no-one ever can. It is a figment of our own imagination.’

Almost a century after Heitler, London and Mulliken, quantum mechanics is still in good health, while chemists continue to use local concepts such as the functional group, the reality of which is supported by decades of accurate additivity rules in experimental thermodynamics (Cohen & Benson, 1993 ▸). As Klaus Ruedenberg has repeatedly stated (Ruedenberg, 1962 ▸): ‘Chemistry needs that atoms be somehow preserved in molecules’. To what extent atoms or functional groups persist in molecules and how they can be extracted from an intrinsically nonseparable molecular wavefunction remains a relevant question in our age of large-scale computations.

Over the past four decades, there has been a significant evolution in our understanding of how the objects of chemistry emerge from quantum mechanics. It has been noted that interpretations of chemical phenomena are highly dependent on the specific computational schemes, levels of theory and other methodological features used in electronic structure calculations. To address this issue, orbital-invariant techniques have gained prominence. These formalisms rely on descriptors derived from reduced density matrices of various orders, whether in real or momentum space. They possess the unique property of being invariant under orbital transformations that keep the wavefunction unchanged. This characteristic makes them versatile and suitable for comparison across various methodologies. Notably, many of these orbital-invariant descriptors have been developed in real space. The simplest of all of them is the electron density itself, ρ, which was used by Richard Bader to build the quantum theory of atoms in molecules (QTAIM) (Bader, 1990 ▸), now one out of many topological approaches to the chemical bond (Martín Pendás & Contreras-García, 2023 ▸). Given that ρ in a crystal is an observable obtained by Fourier summation over all Bragg structure factors, the QTAIM has been widely used to analyze the results of modern high-accuracy X-ray diffraction experiments (Gatti & Macchi, 2012 ▸). The atoms of the QTAIM have been shown to be transferable and to contribute additively to expectation values.

In this realm, many orbital-invariant shell descriptors have been proposed, including the Laplacian of the electron density, ∇^2^ρ, the electron localization function (ELF) of Becke & Edgecombe (1990 ▸) or the localized orbital locator (LOL) of Schmider & Becke (2000 ▸), to name a few. Some of these, like ∇^2^ρ, work well for lighter elements but fail to locate valence shells in heavier atoms. Others, like ELF, resolve all the shells in most of the atoms of the periodic table. When obtained in molecules, these descriptors have provided a wealth of information, and have allowed one to easily discern bonded and non-bonded regions and to confirm that cores remain constant while valences distort considerably upon bond formation. The accumulated knowledge has been used to understand and classify chemical bonds and their change in chemical reactions, and for many researchers this topic is basically closed, needing no further investigation. In recent years, however, a few of us have started to look back at the core of real-space reasoning after noticing several points that deserve clarification. For instance, the positions of the maxima of −∇^2^ρ, LOL and ELF show considerable variations in a given atom. An even larger spread is found in the electron count obtained after integrating ρ in the associated attraction basins of these fields.

In our opinion, much of this state of affairs comes from the loss of information that necessarily follows after compressing the *N*-electron information contained in the wavefunction of a system Ψ into lower-dimensional descriptors like the one- or two-particle densities. We expect that this dimensionality reduction problem that is starting to be unveiled can lead to previously undetected insights. We have recently approached this problem by examining the 3*N*-dimensional maxima of the full Born probability, |Ψ|^2^ (Menéndez-Herrero *et al.*, 2022 ▸). This discretization allows us to bypass the dimensionality curse, since examining the full spatial behavior of a scalar field of 3*N* coordinates is out of the question.

This perspective has garnered support from a number of authors, commencing with Artmann and Zimmerman back in the late 1940s (Artmann, 1946 ▸; Zimmerman & Rysselberghe, 1949 ▸). Their work was buried in the specialized literature for decades due to the computational complexity of the problem, and was rescued by Savin and Scemama in the 2000s (Scemama *et al.*, 2006 ▸) and by Lüchow and Schmidt in the last decade (Lüchow, 2014 ▸; Reuter & Lüchow, 2021 ▸, 2020 ▸; Liu *et al.*, 2020 ▸). All of these authors have shown that the position of the *N* electrons at the |Ψ|^2^ maxima provides lively images of the electronic structure of molecules that are immediately translated into the chemist’s ordinary language. In the aforementioned work (Menéndez-Herrero *et al.*, 2022 ▸), we convincingly showed that the spread of distances at which the atomic shells are observed in the commonly used shell-structure descriptors were due to dimensionality reduction, that the consideration of the full Born probability leads to a non-negligible shrinking of these distances, and that it was −∇^2^ρ, the so-called *L* function, that was the shell descriptor to show better general positional agreement with the Born maximum. The electrons at the maximum of |Ψ|^2^ display exquisitely structured shells in all the cases examined, with geometries that minimize electron repulsions in agreement with Linnett’s (Linnett, 1961 ▸; Luder, 1967 ▸) predictions. Also, upon molecular formation the Born maximum can be usually interpreted as a juxtaposition of atomic distributions that keep their independence, with electrons located at distances from nuclei that expand only ∼10–15% with respect to those found in free atoms in the case of bonding directions.

We take in this contribution the same approach to show, through a guided tour via a set of selected examples, that the electronic structure of atoms is preserved in molecules to a much larger extent than originally expected. Much as in Menéndez-Herrero *et al.* (2022 ▸), the Born maximum is obtained through quantum Monte Carlo calculations. We have computed single-determinant wavefunctions for closed shells, or the simplest symmetry-adapted configuration state functions (CSFs) for open-shell states, and performed variational Monte Carlo (VMC) runs to locate the *N* spin-spatial coordinates maximizing |Ψ|^2^.

Although the computational tools currently available only allow us to explore Born maxima of isolated molecules in the gas phase, nothing prevents the methodology described here from being applied to extended systems. In fact, QMC is fairly standard in periodic systems, but no code has yet been adapted to this particular maximization task. We hope that the ideas we develop below will attract the attention of the crystallographic community, which has successfully adopted many of the tools for analyzing chemical bonds that come from wavefunction analyses (or their reduced densities).

We start by briefly reviewing the methodological and computational aspects of our approach, with a grasp of the more technical aspects relegated to the supporting information. After this, we revisit the Born maximum of atoms, emphasizing how shells and sub-shells appear giving rise to the well known periodic behavior. In this section[Sec sec3], we state a simple rule that predicts reasonably well the geometries found at the computed Born maxima and also consider a couple of examples for 3*d* transition metal ions. The section ends by considering the Born maxima of simple excited atomic states. Section 4 is devoted to showing the persistence of the previously discussed atomic Born maxima in molecules. The role of local maxima close in probability to the global maximum is highlighted and contextualized in terms of the classical concept of promotion. Finally, we join all the pieces together in a case study that reveals the predictive abilities of this approach.

## 2. Methodological and computational details

The 3*N*-dimensional maxima of |Ψ|^2^ have been obtained through the *AMOLQC* suite (Lüchow *et al.*, 2021 ▸). In short, single-determinant wavefunctions using the cc-pVDZ basis set in the case of closed shells or symmetry-adapted intra-shell complete-active-space wavefunctions in the case of open shells have been obtained with the *GAMESS* (Schmidt *et al.*, 1993 ▸) code. In a second step, pure variational quantum Monte Carlo calculations that must reproduce their energies were performed and the coefficients of Jastrow factors to include the effect of electron correlation were optimized, such that Ψ = exp(*U*)Φ. Here Φ is a single determinant or one of our minimal CSFs and *J* = exp(*U*) is the Jastrow factor, where *U* is expanded in terms of explicit interelectron and electron–nucleus coordinates. Details can be found in the supporting information (Section 1). In the course of the VMC runs, a detour as implemented in *AMOLQC* was taken to minimize 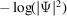 . This was done at equally spaced steps of the sampling procedure, using a combination of steepest descent and L-BFGS minimization algorithms (Lüchow *et al.*, 2021 ▸). The minimization of 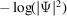 takes advantage of the exponential evolution of electron densities, density matrices and wavefunctions, allowing a single algorithm such as steepest descent search to find maxima of |Ψ|^2^ efficiently in both high and low Born probability, which can differ by many orders of magnitude. Whenever needed, the effect of α–β separation on the Born maximum is examined by comparing the results of with (HF+J) and without (HF) Jastrow optimizations. Details of the specific Jastrows used in this work are found in the supporting information.

Since the qualitative features of single-particle descriptors such as ELF, LOL and *L* do not need high levels of theory, extended basis sets or relativistic effects, they will be obtained, when needed, from the parent non-correlated wavefunctions using our *Promolden* code (Martín Pendás & Francisco, 2023 ▸).

## 3. The Born maximum of atoms: shells, sub-shells, periodic behavior

### 3.1. Ground state of main group elements

The maximum of 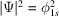 in the H atom places the electron obviously at the nucleus. In the ^1^S ground state of He, the second (opposite spin) electron has no impediment to lie also at the nucleus, and thus the two electrons are placed at *r* = 0 in the Born maximum. This can be immediately seen at the mean-field level, where the wavefunction is the single Slater determinant 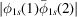 . The square of its spatial part is simply 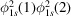 , maximized when the two electrons are at the maximum of the 1*s* orbital. This result also holds for the exact non-relativistic solution. The structure with two electrons at each atomic nucleus, which we can call the K shell or the 1*s* ^2^ core, is maintained for all atoms if described under a standard Coulomb Hamiltonian. In Li and Be, the third and fourth electrons must avoid the nucleus, and are thus located at a given distance from it, *r*. At the HF+J level, these are equal to 1.76 and 1.18 au, respectively. In the absence of electron correlation, *r* decreases as expected (to 1.72 and 1.12 au, respectively). Notice that there are an infinite number of degenerate Born maxima as dictated by the overall spherical symmetry of both systems. This means, for instance, that in Be the two opposite-spin electrons occupy the endpoints of a freely rotating dumbbell that passes through the nucleus. This is the 2*s* ^2^ sub-shell, and as we discussed earlier (Menéndez-Herrero *et al.*, 2022 ▸), the value of *r* at which it occurs is considerably smaller than that obtained from other measures as a result of the multi-electron nature of the Born maximum. For instance, the external maximum of the square of the 2*s* orbital ( 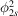 ) has *r* = 1.36 au.

Let us stress that the dumbbell is exclusively due to electron correlation effects, thus being quite labile. At the single-determinant level, α and β electrons are independent, and their positions are uncorrelated. There are two same-spin and one opposite-spin valence electrons in the 1*s* ^2^2*s* ^2^2*p* ^1^-^2^P ground state of B. Pauli exclusion (Fermi correlation) is extremely more intense in separating same-spin electrons than Coulomb correlation in doing the same for opposite-spin pairs. A triangular disposition appears with the same-spin pair at the end of a dumbbell that is slightly perturbed by the opposite-spin electron and, as a consequence, the dumbbell does not pass through the nucleus. This is a real-space analog of the Aufbau and Hund rules. Each subset of same-spin electrons acquires a rigid structure that minimizes their mutual Pauli repulsions, while the relative position of α and β blocks is considerably more labile.

**Figure 1:**
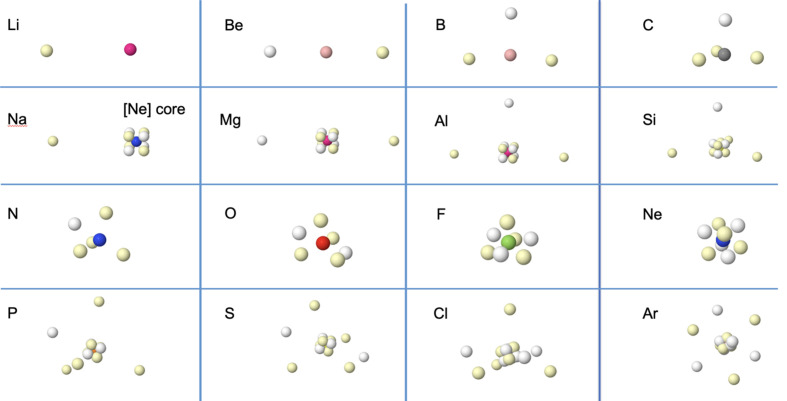
Ground-state HF+J Born maxima for atoms Li to Ar. Different spin electrons are colored in white and yellow. The inner (1*s* ^2^) atomic cores, displayed as colored spheres following a standard CPK coloring scheme, contain two hidden opposite-spin electrons located at the nuclei. Second-period atoms are shown in the first and third rows, while third-period atoms occupy the second and fourth rows, so that same-group atoms are stacked columnwise and the periodicity described in the main text is clearly uncovered. In Si–Ar, the outer core hides the inner core at the scale of the plot.

The buildup of the periodic behavior in the Born maxima of atoms is summarized in Fig. 1 ▸. A simple two-step thumb rule to understand proceeds as follows. (i) Count the number of valence α and β electrons and place them, separately, on the surface of a sphere, building a polyhedron for each block that minimizes their Pauli repulsion. This is equivalent to Thomson’s problem regarding the minimum energy distribution of a number of equivalent charges on a spherical surface (Thomson, 1904 ▸). (ii) Rigidly rotate these two polytopes around each other to minimize their Coulombic repulsion. In ^3^P carbon or ^4^S nitrogen this procedure leads to a majority spin triangle or tetrahedron, respectively, to which a single minority spin electron is added leading to the geometries found in the figure. Upon reaching the Ne atom, it is quite obvious that the two equivalent tetrahedra will be placed forming a perfect cube. Summarizing, an *ns* ^2^ sub-shell gives rise to a dumbbell and an *ns* ^2^ *np* ^6^ shell (or sub-shell) gives rise to a cube.

If we move from Ne to Na, the cubic 2*s* ^2^2*p* ^6^ L shell does not admit any further electrons, so the new one occupies another shell. If we zoom out, ignoring the [Ne] core, the Born maxima of Na–Ar replicate the geometries found for Li–Ne, and the textbook periodicity sets in. A delicate fine structure is visible: for instance, the extra electron in Na is located on top of the center of one face of the [Ne] core cube to minimize repulsions.

**Figure 2:**
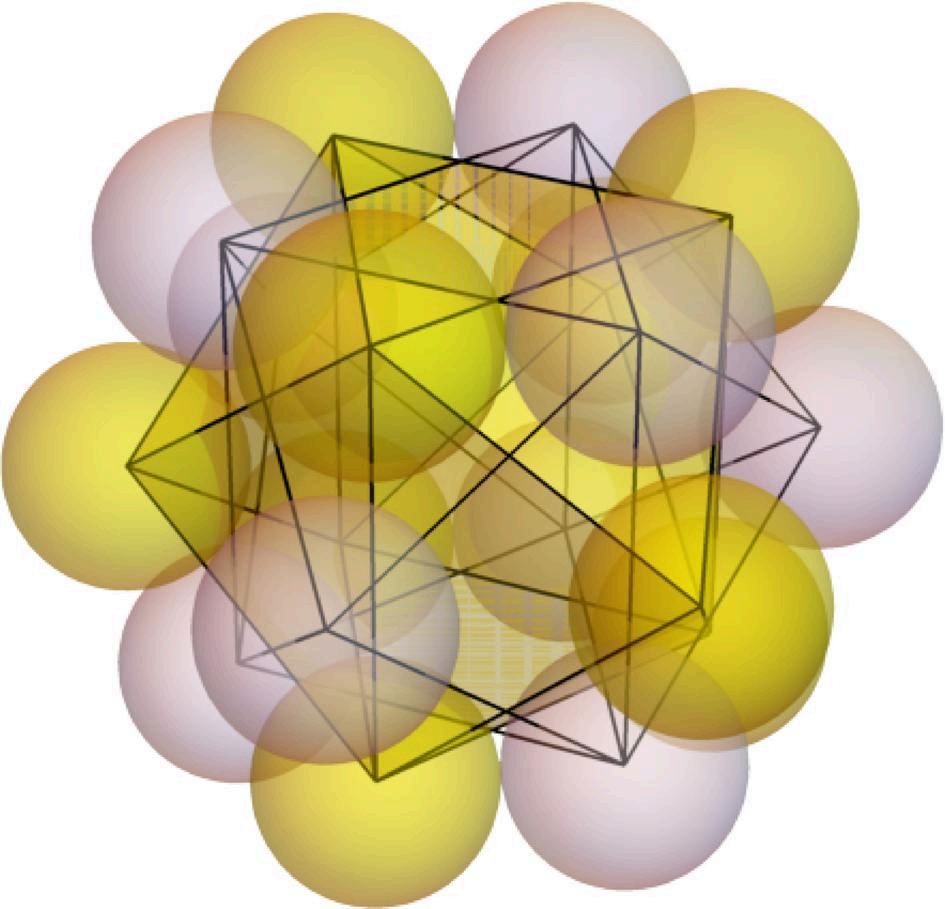
The two interpenetrated nine-vertex polyhedra forming the 18-electron valence shell in Kr. Different-spin electrons are displayed in white and yellow. HF+J results.

The structure of the 18-electron *ns* ^2^ *np* ^6^ *nd* ^10^ shell has already been reported (Menéndez-Herrero *et al.*, 2022 ▸). It is formed by two equivalent polyhedra with nine same-spin electrons, each of them adopting the well known configuration of nine-ligand complexes like ReH 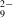 . It is described as either a capped square antiprism or a tricapped trigonal prism. These two polyhedra interpenetrate each other to form a quasi-spherical object with three hexagonal close-packed layers of alternating α and β electrons, as shown in Fig. 2 ▸

### 3.2. Ground state of selected transition metal elements

**Figure 3:**
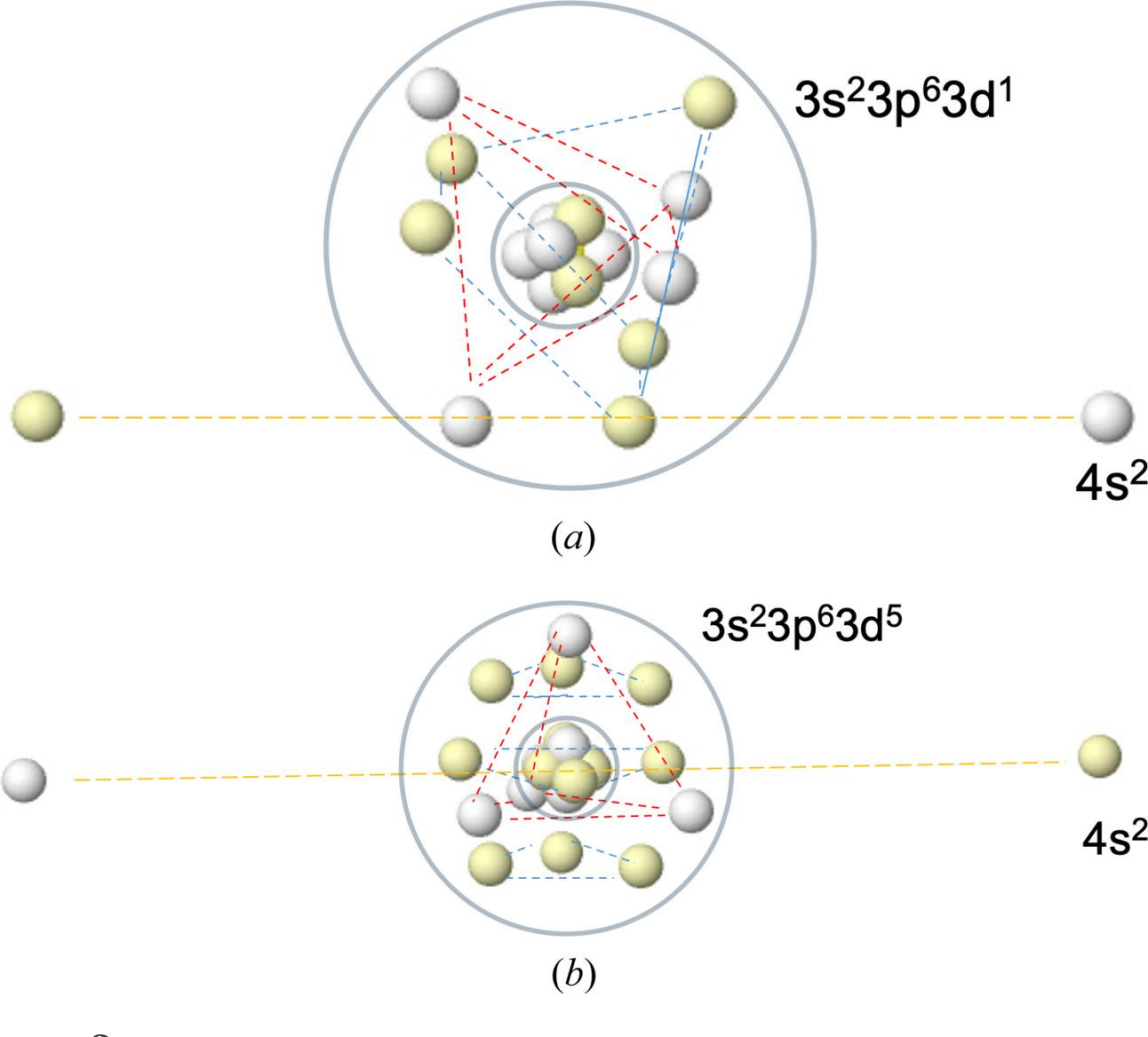
The HF+J maxima of the ^2^D state of Sc (top) and the ^6^S state of Mn (bottom).

The Born maxima of the K and Ca atoms in their ground states are analogous to those of Na and Mg in Fig. 1 ▸ if we substitute the [Ne] core with an [Ar] one. In agreement with what we teach in fresh chemistry courses, the addition of extra electrons induces the filling of the M shell from Sc to Zn. In this process, we pass from an eight-electron cube to the previously described 18-electron polyhedron. Since we do not intend to be exhaustive, we will present just two examples, the ^2^D state of Sc and the ^6^S state of Mn. Fig. 3 ▸ summarizes our results.

In Sc, the M shell has five α and four β electrons. Although the α set would, in principle, be predicted to adopt a trigonal bipyramid geometry, we find a square pyramidal geometry instead, probably due to the influence of the β set. More accurate calculations could change this, although the pattern reported here was found to be rather stable. As expected, the tetrahedral structure of the minority spin block is very robust, and both polyhedra rotate until minimal repulsion is found. Interestingly, the pair of electrons that we would associate with the 4*s* ^2^ N sub-shell form a dumbbell that does not pass through the nucleus. This is obviously the result of the net dipole moment of the nine M shell electrons, or using a different language, of the non-zero angular momentum of the D eigenstate. Notably, the ground state of ScH_2_ is non-linear with an H—Sc—H angle of ∼133° (Balasubramanian, 1987 ▸), to be compared with the ∼145° angle formed by the two N electrons and the nucleus in the figure. We will later relate this to the angular geometry of heavier alkaline-earth halides or hydrides.

In contrast, the ground state of manganese is a sextet with vanishing angular momentum. The majority spin block of the M shell is full (nine α electrons), while the minority spin block retains as many electrons as in scandium. In Fig. 3 ▸ we can check that the first block adopts the previously described capped square antiprism geometry, while surely the second block forms a tetrahedron. Both sets have a null dipole and nicely interpenetrate with minimum Coulombic repulsions. The 4*s* ^2^ N dumbbell is symmetric and passes through the nucleus.

### 3.3. Excited states

Born maxima can also be obtained for excited states. We find it useful in the following to discuss the simplest intraconfigurational excitations of carbon, nitrogen and oxygen. In the case of carbon, we also add 1*s* ^2^2*s* ^1^2*p* ^3^ ^5^S, which is invoked many times in the promotion of the C atom to an appropriate tetravalent valence state.

**Figure 4:**
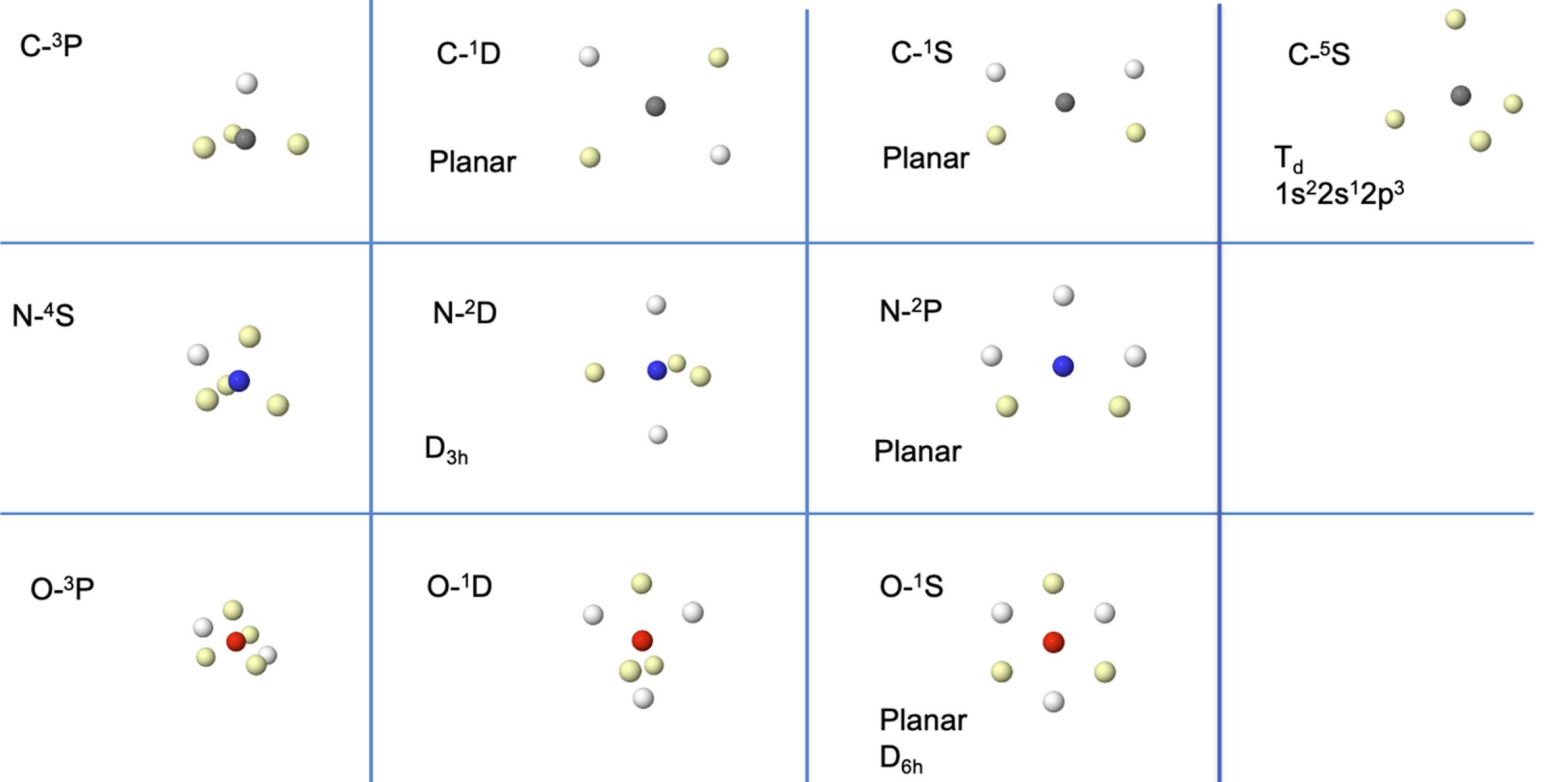
Born maxima of the *p* ^2^, *p* ^3^ and *p* ^4^ Russell–Saunders states of C, N and O. The first interconfigurational ^5^S state of C is also shown. HF+J results.

Fig. 4 ▸ shows the Born maxima of these *p* ^2^, *p* ^3^ and *p* ^4^ Russell–Saunders states. The left column displays the ground state already discussed.

The first surprising fact concerns the two-dimensional nature of many of the electron arrangements. This clashes with the simple electron-repulsion rules that work so well for ground states, and reinforces the idea that excited-state chemistry does not necessarily follow the rules and regularities we grow up with. Surely, models like Gillespie’s valence-shell electron-pair repulsion (VSEPR) theory (Gillespie & Hargittai, 2012 ▸) require adjustment in these cases, to say the least. In C, for instance, both the ^1^D and ^1^S states display the four valence electrons on a plane. In the first case, the two same-spin electrons occupy the diagonals of the rectangle, while in the second case it is the opposite-spin electrons. Not unexpectedly, if the four same-shell electrons in the ^5^S state are forced to be unpaired, a perfect tetrahedron is found.

In N, both the ^2^D and ^2^P states have a 3 + 2 electron count. The first follows our rules of thumb, forming a perfect triangular bipyramid, while the second is completely planar, with a distorted pentagonal geometry. Finally, in oxygen, the 3 + 3 ^1^D state consists of two equilateral same-spin triangles rotated 90° with respect to each other and the ^1^S state is a perfectly planar alternating hexagon. As we will see, these structures can be found in molecules in which they make perfect chemical sense.

## 4. Persistence of the atomic structure in molecules

### 4.1. Recognizing atoms in molecules

Born maxima have been reported several times in molecules (Lüchow, 2014 ▸; Liu *et al.*, 2020 ▸). Let us just show how the atomic structure can immediately be read out from them in a couple of examples.

**Figure 5:**
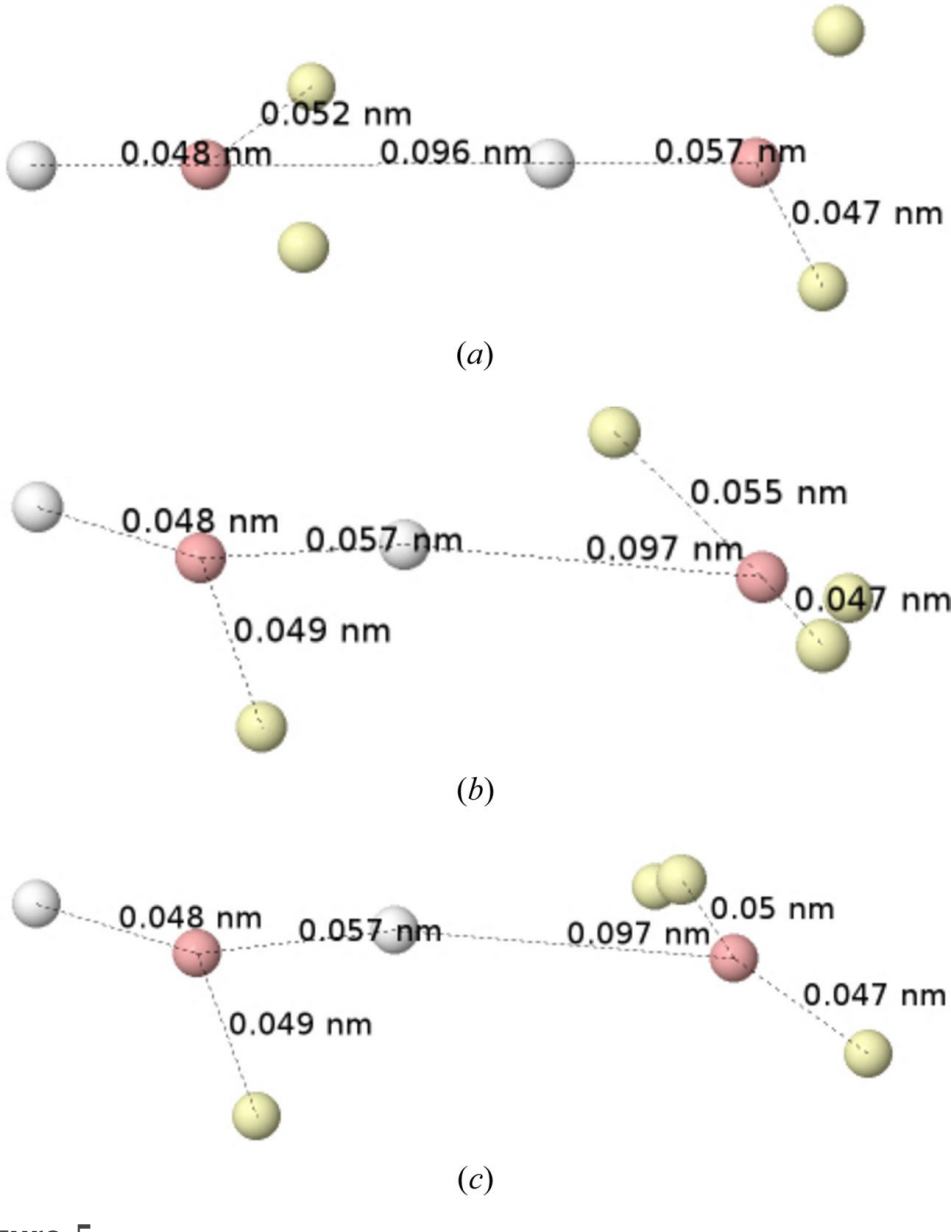
Born maxima of the 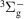 ground state of B_2_. Global maximum (top). Two local maxima showing spin excitations at the right-most B atom (middle and bottom). HF+J results. Several relevant distances are also shown.

As is well known, the presence of multiple nuclei opens up additional electron-distribution possibilities around each center and a number of local maxima can occur in addition to the global one. Figs. 5 ▸ and 6 ▸ display the global and several local maxima of |Ψ|^2^ in the ground state of B_2_ ( 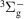 ) and C_2_ ( 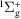 ), respectively. Two independent atoms are effortlessly recognized. At the global maximum, each of the two B atoms is also identified as having the same planar electron distribution as a ground-state ^2^P B (see Fig. 1 ▸), in two orthogonal planes. The relation of this to bonding is extremely important but beyond the scope of this work. In the lower panels, two lower-probability local maxima are shown, in which one of the atoms (the left-most one) has been excited to a ^4^P state, with all the valence electrons unpaired. Again, this finding is directly related to the fact that an atom in a molecule is an open quantum subsystem (Pendás & Francisco, 2019 ▸) coupled to its environment, but again we will not pursue this further. In all the maxima, spin-coupling rules apply easily. The triplet is formed either by the coupling of ^2^P atoms in the global maximum or by a ^2^P × ^4^P coupling in the local maxima.

**Figure 6:**
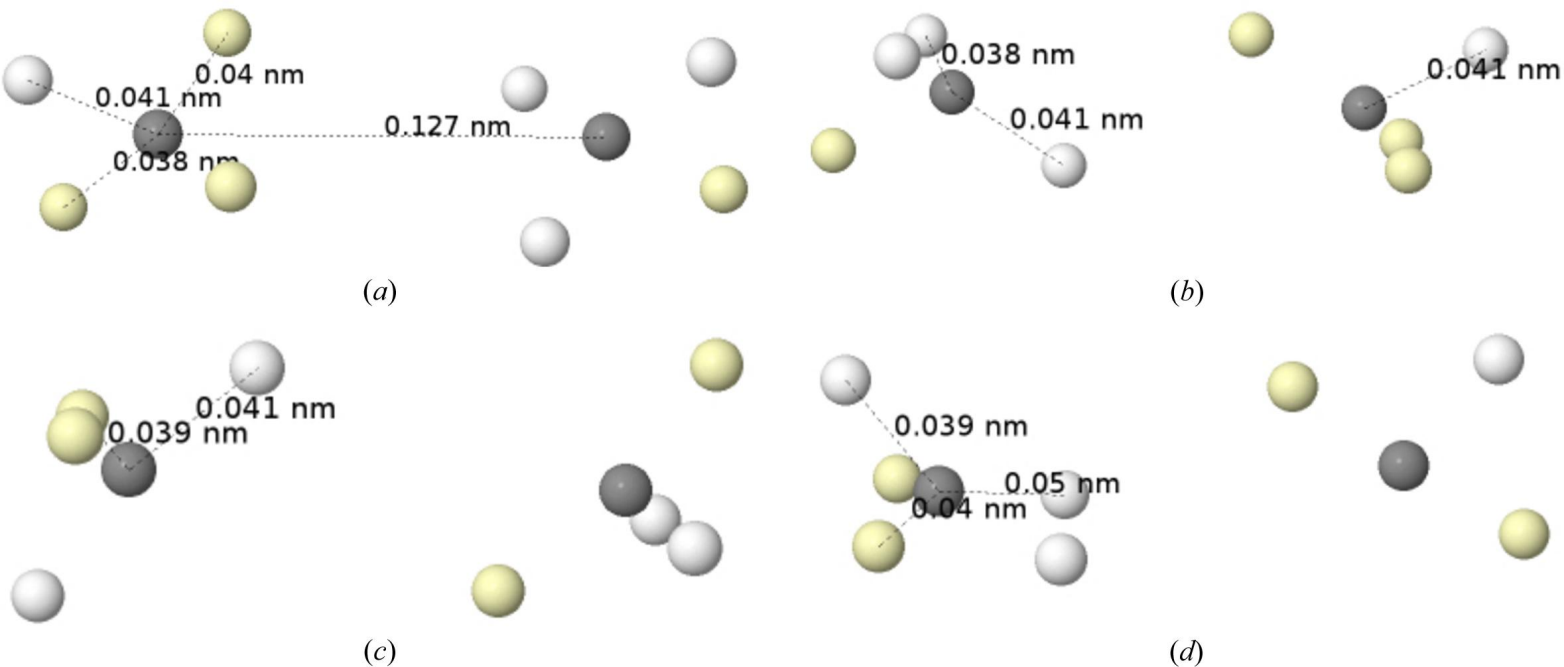
Born maxima of the 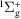 ground state of C_2_. Global maximum (top left). Rotated local maximum (top right). Spin-excited and ionic local maxima (bottom left and right, respectively). HF+J results. Several relevant distances are also shown.

The same principles apply to the dicarbon molecule. The ground state is formed by the coupling of two clearly isolated ^3^P atoms in, say, *M*_*s*_ = ±1 states. Several local maxima with simple rotations of one of the atomic distributions are also found. In the lower left panel of Fig. 6 ▸ we recognize two ^1^D C atoms coupled to a singlet (see Fig. 4 ▸), with almost planar electronic distributions. In the lower right panel, we see an ionic structure in which the left C atom has an extra electron and is isoelectronic with an N atom in the ^2^P state, and the right C atom is a cation isoelectronic with the ^2^P state of B. Spin-coupling rules are obviously fulfilled.

In summary, atoms are clearly preserved in molecules, with electronic states and electron counts that vary in accordance with their open quantum mechanical nature. Moreover, atoms in molecules can be found in promoted (excited) electronic states, consistent with conventional wisdom. We will examine this in more detail in the next subsection.

### 4.2. Promoted atoms in molecules

#### 4.2.1. The water molecule

**Figure 7:**
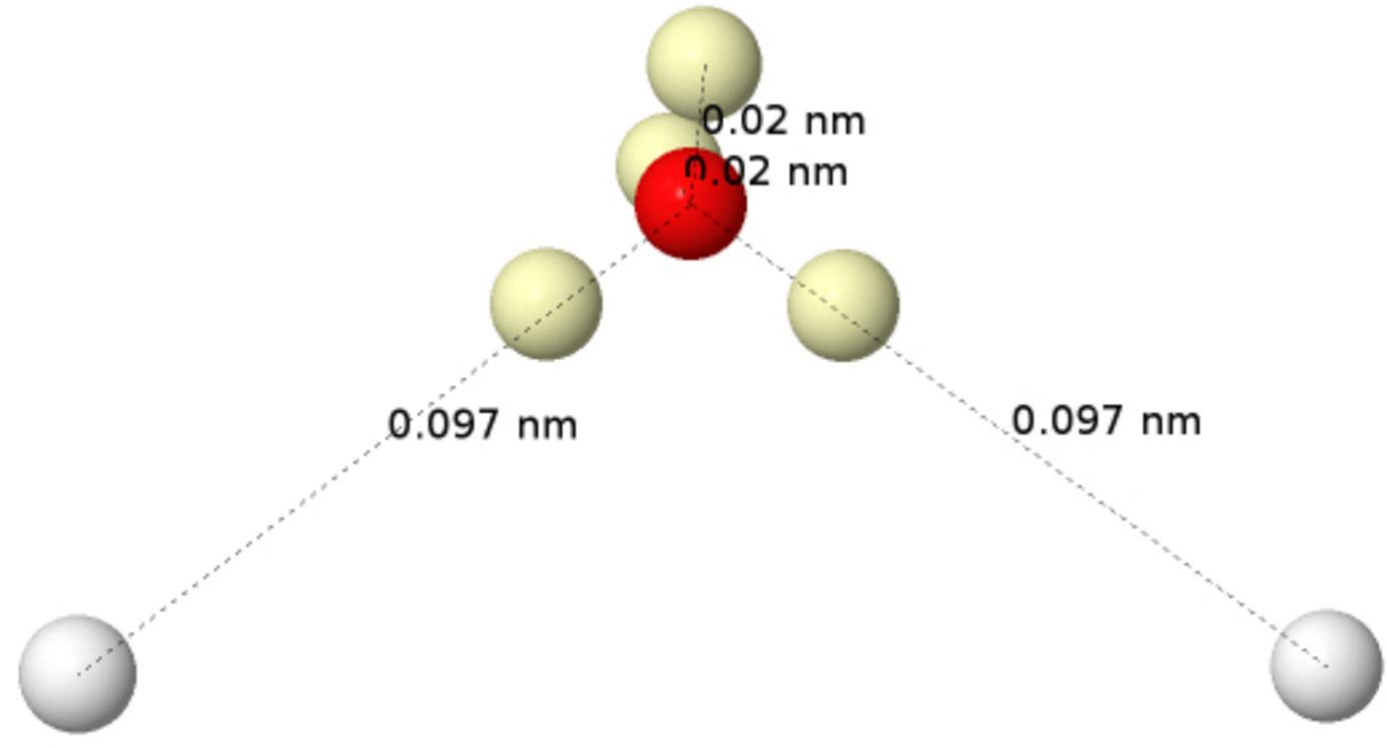
The Born maximum in the ground state of H_2_O at the HF level. The four yellow spots are coalesced α–β pairs.

We will examine three illustrative simple cases. Water is one of the paradigmatic systems where real-space techniques show their superiority over conventional orbital approaches. Since its electronic structure is well described by a single determinant, textbooks teach how, in a minimal canonical basis description, a purely nonbonding 1b_2_ function (the oxygen 2*p*_*x*_ orbital) together with a slightly bonding 3a_1_ orbital are interpreted as the π and σ components of two lone pairs (LPs). Although rabbit-ear LPs can be obtained by orbital localization techniques, any of the *L*, ELF or LOL scalar fields will easily recover the two equivalent electron-pair localization regions. The Born maximum at the HF level, Fig. 7 ▸, shows an ionic structure with four valence α–β coalesced pairs in a tetrahedral arrangement around the oxygen atom at positions in good agreement with those found from the minus Laplacian.

**Figure 8:**
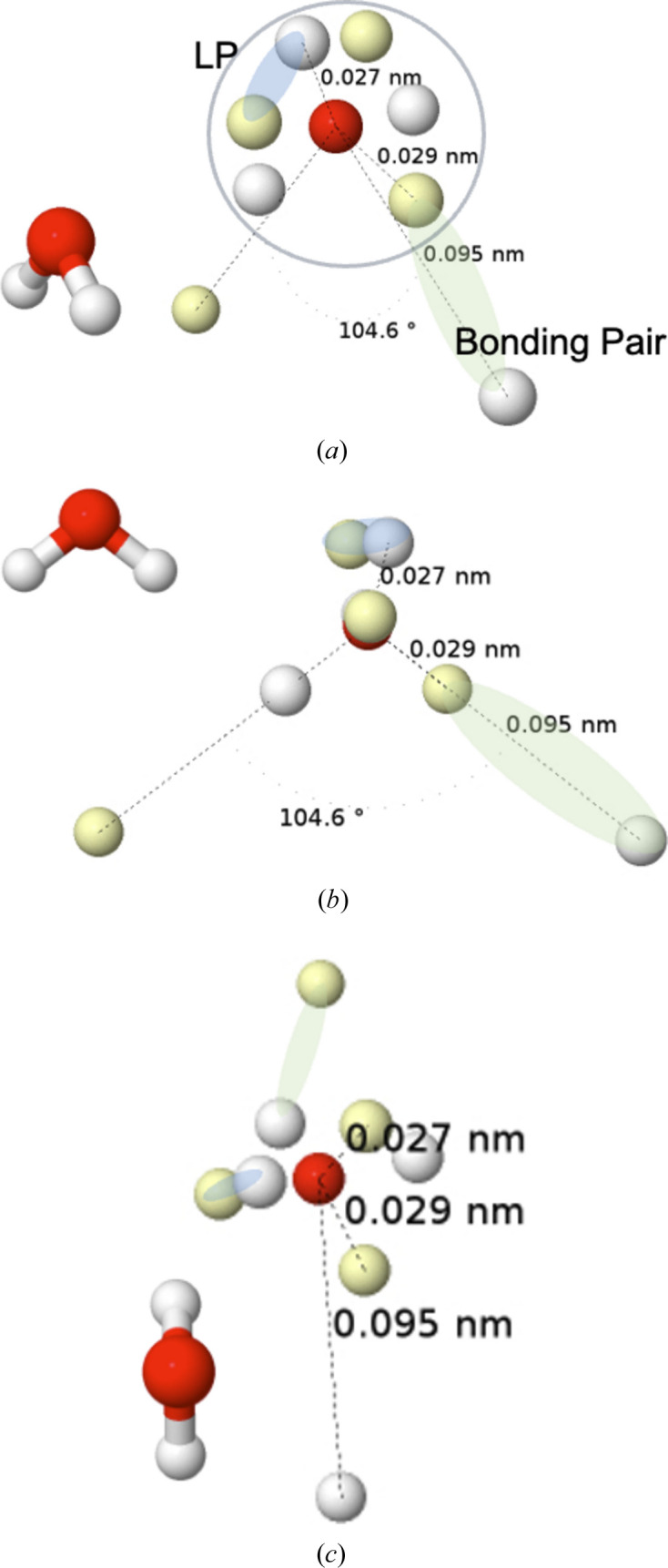
The HF+J Born maximum in the ground state of H_2_O in three different perspectives. A representative of each of the two lone and bonding pairs is highlighted in cyan and light green, respectively.

The introduction of Coulomb electron correlation separates the pairs, and the HF+J global maximum is shown in Fig. 8 ▸, in several perspectives. The maximum displays one electron at each of the H nuclei, with different spin projections, and six electrons around the oxygen nucleus, four of them at an equivalent slightly shorter distance than the other two. The latter are spin-coupled to the electrons at the H nuclei, so they can be identified with the two Lewis bonding pairs (BPs). One representative has been highlighted in light green in the figure. Similarly, the remaining four electrons around the oxygen that oppose the hydrogens form two LPs, although the spatial requisites blur the traditional rabbit-ears image considerably. In fact, as we can see in the middle panel of the figure, the position of the rabbit-ear LPs is a kind of average of that of their two constituent electrons, in accordance with the dimensionality reduction that takes us from the multidimensional Born representation to the one-particle picture provided by *e.g.* the *L* function.

It is now crystal clear that there are two sets of rather rigid α and β trios of electrons around the oxygen atom. Using our previous results (see Fig. 4 ▸), their disposition can be described as a mixture between those of the ^1^D and ^1^S states. Definitely an excited singlet, not a ground-state triplet oxygen atom. Interestingly, the bottom panel of Fig. 8 ▸ shows how the axis of each of the two BPs is shifted by ∼20° with respect to the geometric O—H direction. The observed geometry is a compromise between bonding requirements and the energetic penalty of distorting the atomic structure of the oxygen atom. Once again we see how well the electronic structure of atoms, this time in an excited state energetically close to the basal state, can be recognized in molecules.

These results are in excellent agreement with ideas coming from other branches of real-space analyses. For example, in the interacting quantum atoms approach (IQA; Francisco *et al.*, 2006 ▸), each atom in a molecule is endowed with a self-energy that tends toward the free atomic energy as its interactions with the environment vanish. It is well known that the self-energy of an atom increases during bonding, so there is an energy penalty (the so-called deformation energy), which is more than paid by the new attractive forces established during the process. These deformation energies have been associated with classical (Menéndez-Crespo *et al.*, 2018 ▸) promotions. We believe that this interpretation is reinforced by the results described here.

Finally, it is relevant to highlight that typical textbook models considering bonding in water fail to assign the most probable spin arrangement found in the BPs. Whether we use a ground-state ^3^P oxygen (2*s* ^2^2*p* ^4^) or an *sp* ^3^ hybridized one, the two bonding electrons share the same spin projection, and so this spin equality is inherited by the hydrogen bonding electrons in textbook descriptions. This is clearly not correct at the Born maximum and leads to spin–spin correlations that could be measurable.

#### 4.2.2. CH_2_

Our next example is methylene (or methylidene), the simplest carbene. Given its C_2*v*_ geometry, its canonical molecular orbital diagram is very close to that of water. Here, the proximity of the 1b_2_ and 3a_1_ orbitals leads to a triplet ^3^B_1_ ground state, with two unpaired electrons populating these functions. Accurate calculations find an excited ^1^A_1_ singlet 9.0 kcal mol^−1^ above the triplet (Herzberg & Johns, 1971 ▸). Heavier analogs in Si and Ge show an inverted singlet–triplet ordering and have led to very interesting chemistries (Boehme & Frenking, 1996 ▸).

**Figure 9:**
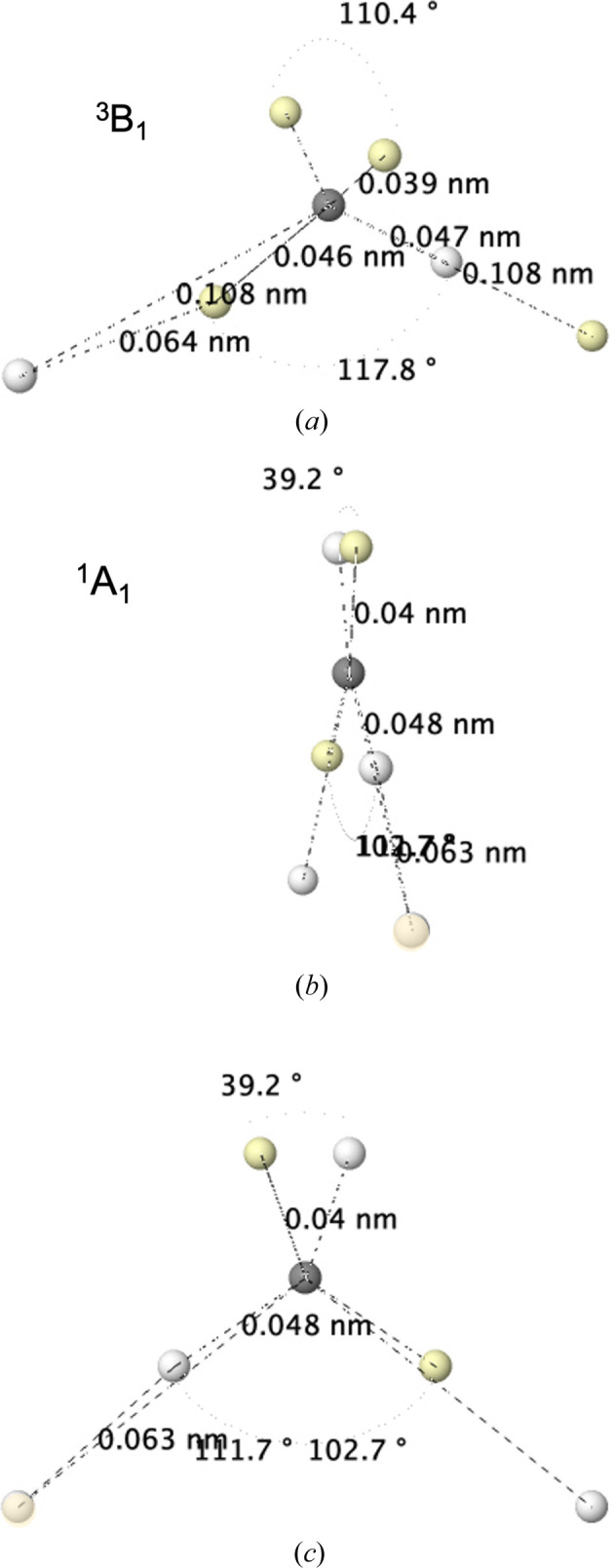
The HF+J Born maximum in the ^3^B_1_ ground (top) and ^1^A_1_ singlet (middle and bottom) first excited states of CH_2_.

Fig. 9 ▸ shows the Born maximum for the triplet and singlet states. From the experience gained in previous sections, the triplet reveals a well developed ground-state carbon atom, with the usual slightly lengthened and shortened electron positions along the bonding and nonbonding directions, respectively.

The system is immediately imagined as a rabbit-ear diradical, with each LP containing an electron. The spin coupling is always exquisitely obeyed, and again the two H atoms carry different spin-projection electrons.

The middle and bottom parts of Fig. 9 ▸ depict the singlet excited state. The four electrons around the carbon atom are located on a plane, with an LP that closely corresponds to the doubly occupied 3a_1_ function in conventional molecular orbital theory. Comparison with Fig. 4 ▸ tells us that this is a ^1^D carbon, in a ^1^D × ^2^S × ^2^S coupling with the two H atoms. Not unexpectedly, the angle between the two electrons forming the diradical is considerably larger than that forming the LP in the singlet.

#### 4.2.3. C_3_O_2_

We will conclude our discussion of promoted atoms in molecules by considering a less academic, probably more interesting, case. Carbon suboxide, C_3_O_2_, has a non-linear structure that has been rationalized (Frenking & Tonner, 2009 ▸) in terms of a new category of zero-valent carbon compounds bearing two LPs on a C atom, the so-called carbones. Analogs of heavier elements have also been synthesized. According to this interpretation, two carbonyl groups act as sigma donors via dative bonds to an otherwise spectator central carbon atom. The presence of the two LPs justifies the angular geometry of the system.

**Figure 10:**
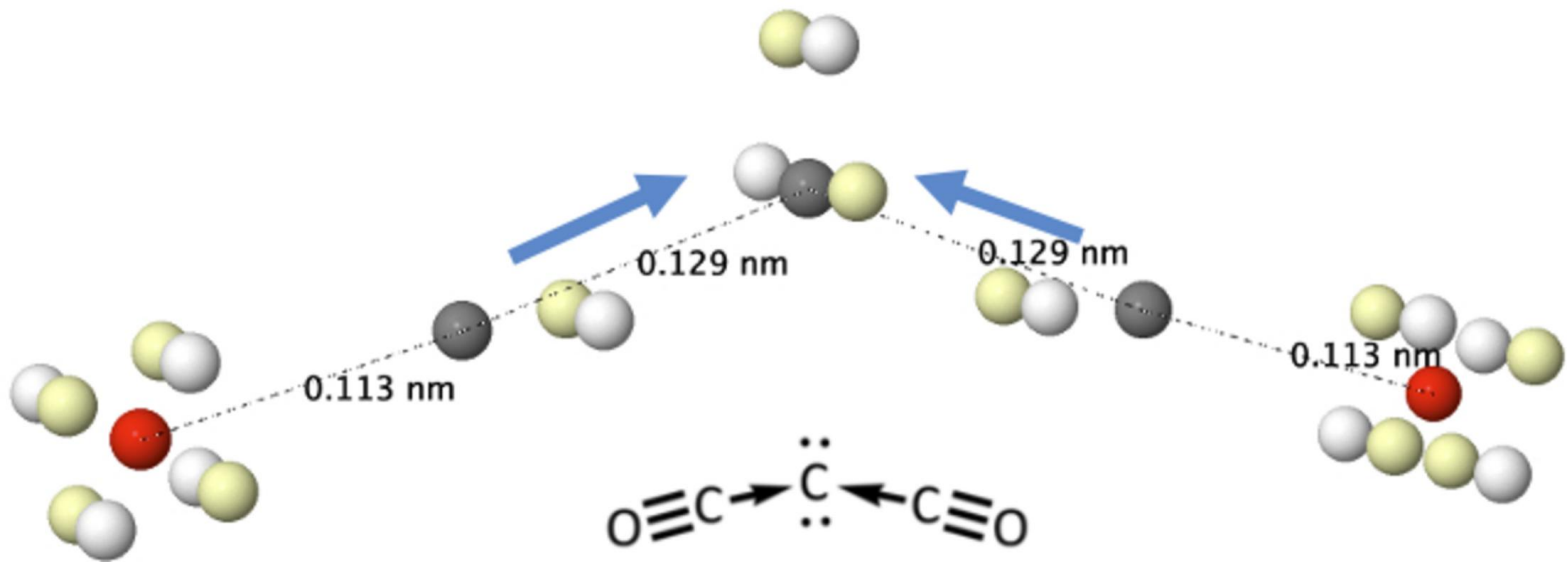
The HF+J Born maximum in the ground state of C_3_O_2_.

The Born maximum image provides a visually impressive confirmation of these insights. Any chemist can read Fig. 10 ▸ directly. Notwithstanding the interesting oxide nature of the oxygen of each carbon moiety, the two dative pairs are easily discerned and, again, the four electrons around the central carbon atoms display the planar structure corresponding to the ^1^D state of C. Its two LPs are also immediately recognized in the plane orthogonal to that of the molecule.

### 4.3. Ionic ground states

**Figure 11:**
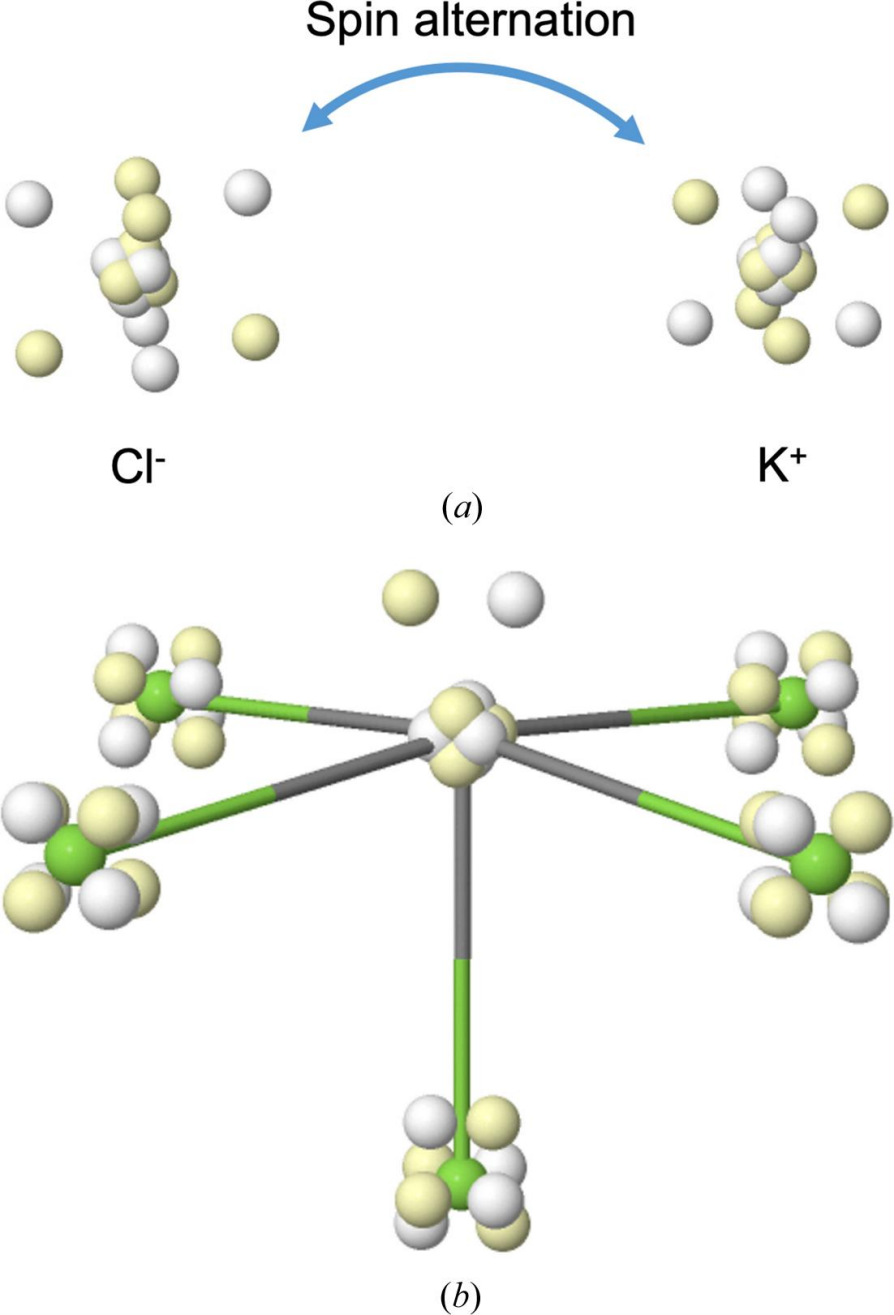
The HF+J Born maximum in the ground state of KCl (top) and ClF_5_ (bottom).

A limiting case of atomic promotion is full ionization, which is obviously expected in classical ionic compounds. Let us show only two simple examples. Fig. 11 ▸ shows the Born maxima of KCl and ClF_5_. As can be seen, the two atoms in KCl are closed shells that preserve the electronic structure of the ground states of the K^+^ cation and the Cl^−^ anion. This fact carries over to ∇^2^ρ, for example, which shows a positive value in the internuclear region and no hint of the N shell around the K atom. In addition, the Born maximum reveals much finer details that remain hidden in a coarse-grained one-particle picture. As shown in the figure, the two valence M shells of K^+^ and Cl^−^ face each other along the edges of the cubes, with a good spin alternation.

Another example is that of classically hypervalent compounds such as ClF_5_. There is broad consensus that pure hypervalency, which in such compounds would imply *d* orbital participation, does not exist (Reed & Schleyer, 1990 ▸), and real-space techniques have shown that ionic participation is very important. For instance, a B3LYP/cc-pVTZ QTAIM analysis shows polarized closed-shell fluorine atoms exhibiting considerably negative charges (−0.93 and −0.97 electrons in the equatorial and apical F atoms, respectively) for the ligands and a ∇^2^ρ with an LP in *trans* geometry with the apical fluorine, in very good agreement with the predictions of the VSEPR model for an AL_5_E_1_ system, with six electron-pair regions around the central atom.

The bottom panel of Fig. 11 ▸ maps these one-particle averages to actual electron positions at the Born maximum. The five F atoms are fluorides with a cubic L shell. These five cubes are oriented such that a pair of spin-coupled electrons forming one of their edges points toward the Cl atom. In this sense, this situation can plausibly be described in terms of dative bonding. The chlorine atom exhibits a textbook LP, and simple electron counting would assign it a formal oxidation number of +5: only two valence electrons surround the Cl atom, in reasonable agreement with a QTAIM charge of +4.81 au.

This is certainly not the end of the story, since the shared-electron bond order between the Cl and F atoms, as measured by the delocalization index, provides relatively large values (0.60 and 0.41 for the equatorial and apical bonds, respectively) that indicate non-negligible Cl–F covalency. A probabilistic analysis, in line with the open quantum systems perspective (Pendás & Francisco, 2019 ▸), is beyond the scope of this work, but it suffices for our purposes here to notice that there are several local Born maxima in which one or a few of the F atoms exhibit ground-state ^2^P configurations with nine electrons and in which the Cl center accumulates up to eight valence electrons.

## 5. Joining pieces in a case study

We devote this section to showing what kind of chemical insights can be obtained from the analysis of Born maxima. We choose a very well studied old problem: the geometry of *AX*_2_ compounds, where *A* is an alkaline earth metal and *X* is a halide or hydride. These are linear systems in the case of Be and Mg compounds but bent systems when *A* = Ca, Sr or Ba. Historically, this problem led Gillespie to revisit his VSEPR model (Gillespie, 1992 ▸) to include the effect of ligand close-packing. In the real-space realm, the analysis of the Laplacian of the bent compounds (Bader, 2000 ▸) led to the discovery of the so-called ligand-opposed (or ligand-induced) charge concentrations (LOCCs, LICCs). It was found that the usual valence shell charge concentrations that typically appear along the bond directions in main-group element compounds were directed against the ligands in these systems. Over the years, this was found to be the rule when transition metal complexes began to be analyzed, and the same kind of LOCCs were also found when using other scalar fields that reveal pair localization, like the ELF function (Gillespie *et al.*, 2004 ▸).

The transition from linear (VSEPR) to non-linear (non-VSEPR) geometries on moving from Mg to Ca was soon related to the availability of low-lying *d* orbitals that begins with Ca, as previously discussed with hypervalent compounds. Natural bond orbital calculations, for instance, show considerable *d* participation in CaH_2_, but no hints of it in MgH_2_. An interesting discussion of the factors that affect the emergence of non-VSEPR structures in formally *d* ^0^ systems was provided by Kaupp (2001 ▸). Furthermore, σ-bonding (*d* participation) as well as an increasing role of core polarization as the polarizability of the metal center grows favors bent structures, while ligand repulsion and π-bonding stabilize linear arrangements. The role of the polarizability of the metal has been recently revised, and Broer and coworkers (Linker *et al.*, 2020 ▸) have convincingly shown that simple quasi-classical calculations based on an extended Debye polarizability model are able to systematize the experimental geometries.

**Figure 12:**
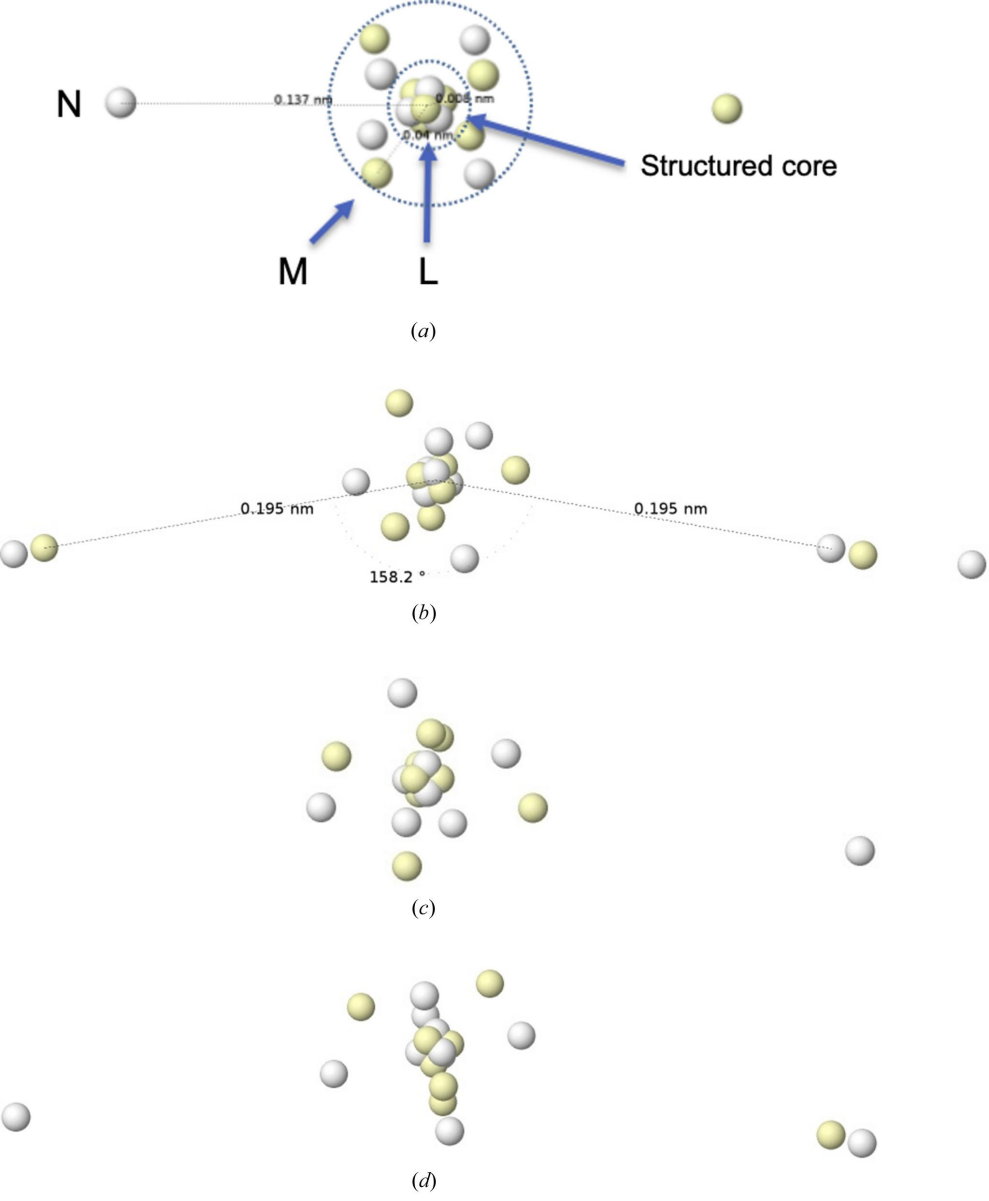
HF+J Born maxima. From top to bottom: ^1^S ground state of Ca, global maximum in CaH_2_, mono-ionic local maximum of CaH_2_ and di-ionic local maximum of CaH_2_.

In Fig. 12 ▸ we gather some of our results on the ground states of the Ca atom and the CaH_2_ molecule, which is clearly bent at the M06-2X/cc-pVDZ level of theory, with *r*(Ca—H) = 2.04 Å, a bond angle of 158° and a rather hydridic character, with a QTAIM charge for the H atom equal to −0.80 au.

First, we observe that Ca has its expected Born maximum. The N shell consists of a dumbbell of electrons with opposite spins, which are placed at a distance of *r* = 1.37 Å from the nucleus by cutting the M cube through the center of two opposite faces. Interestingly, the L shell is also structured on the basis of the M shell. Consistent with the high negative topological charge of the H atoms, the global maximum of the CaH_2_ molecule is ionic, with each H atom carrying two electrons.

From the point of view of the maxima of |Ψ|^2^, the formation of the CaH_2_ system can be seen as the capture of the two outer N electrons of Ca by two H nuclei. In this process, the Ca moiety becomes a Ca^2+^ cation and each H becomes a hydride. If we stop at this point, there is no clear reason for the bending. However, an examination of the local maxima of close probability tells a different story. In the bottom two panels of Fig. 12 ▸ we show two of these, where either one or both H atoms are neutral, and the associated electrons are now clearly in the Ca influence region. Most importantly, these electrons are found at about the same distance from the Ca nucleus as the rest of the electrons previously identified as M electrons. In other words, they can be understood as entering the 3*d* subshell of Ca (there are no angular-momentum distinctions in Born maxima). This has an interesting reading: under the influence of the field produced by the H nuclei, the valence shell of Ca becomes unstable, and their electrons lie either as hydride H electrons or as M-shell Ca electrons.

This phenomenon is nothing more than another case of atomic promotion. As in our previous cases, the persistence of the Born maxima is remarkable. In the mono-ionic maximum, the 5α + 4β system around the Ca atom is isoelectronic with ^2^D scandium (see Fig. 3 ▸), so we are effectively observing a ^2^S(H) × ^2^D(Ca)^+^ × ^1^S(H)^−^ coupling. Similarly, the di-ionic maximum shows a 5α + 5β structure around a 3*s* ^2^3*p* ^6^3*d* ^2^ Ca, isoelectronic with one of the singlet intraconfigurational excited states of the Ti atom and formed by two interpenetrated trigonal bipyramids.

At this point, we return to our discussion in Section 3.2, where the non-vanishing dipole moment of the Born maximum of Sc was discussed and related to the distortion of the N-shell electron dumbbell 4*s* ^2^ and the angular geometry of the ScH_2_ molecule. Although in CaH_2_ the overall Born maximum is ionic and symmetric, the non-negligible probability of observing, for example, the asymmetric mono-ionic distribution induces an electrostatically driven bending force commanded by the Ca^+^ dipole moment. Since the dipole maxima are not the global maxima, the bending finally observed is smaller than in ScH_2_.

**Figure 13:**
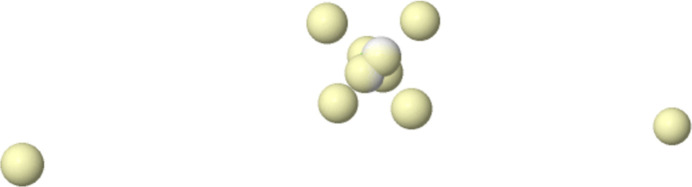
The HF Born maximum in CaH_2_. The cube of the M shell has coalesced into a ligand-opposed tetrahedron of Lewis pairs.

As clearly shown in the H_2_O molecule, electron correlation forces the spatial separation of Lewis pairs, although same-spin blocks remain much more rigid than opposite-spin blocks. In the present case, the M shell of Ca in the global Born maximum does not show any clear indication of LOCCs. However, if we remain at the single-determinant level, the pairs coalesce in the global Born maximum (as also happens in the water molecule). Fig. 13 ▸ reveals that the cube forming the M shell coalesces into a tetrahedron of Lewis pairs that is perfectly ligand opposed. It is thus upon dimensionality reduction that the correlated fine structure of the correlated maximum yields to a mean-field description in which an average is observed. To what extent this fact has an impact on the knowledge accumulated around LOCCs (Mcgrady *et al.*, 2005 ▸) remains to be determined. After all, LOCCs have been rationalized as the response of the valence electrons of transition metals to heavily charged ligands in an attempt to minimize electrostatic repulsion. We plan to perform detailed IQA calculations to shed some light on this interesting problem.

**Figure 14:**
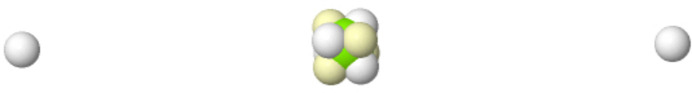
The HF+J Born maximum in MgH_2_.

It is also instructive to consider MgH_2_, which is clearly linear. The HF+J Born maximum is shown in Fig. 14 ▸. No other local maxima were found. We think this supports our instability model, since the mono- or di-ionic configurations would imply more than eight electrons in the Mg L shell, which is not possible.

## 6. Relation to other approaches

At this point it is pertinent, especially with respect to crystallography, to comment briefly on how these results relate to other approaches. Since Born maxima show electrons in real space, we restrict ourselves to spatial methodologies. Roughly speaking, all real-space techniques devised so far to access an atom in a molecule can be unified using a general formalism based on weight functions (Pendás *et al.*, 2007 ▸). Succinctly, at each point in space 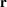 , each atom *A* of a system provides a weight 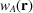 such that 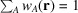 . The weight measures the atom’s share at a particular position. When all atoms contribute a nonzero weight at 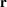 the partitioning is called fuzzy, and when only one does so, exhaustive. The former category includes, for instance, the Hirshfeld or Stockholder partitioning (Hirshfeld, 1977 ▸), which is also the basis of the Hirshfeld atom refinement method (Capelli *et al.*, 2014 ▸). A prominent example of exhaustive decompositions is the QTAIM (Bader, 1990 ▸).

Although the use of Born maxima does not itself lead to an atomic decomposition, our results show that the picture they provide is more compatible with exhaustive than with fuzzy atomic definitions. The persistence of Born maxima is in line with the well documented transferability of the QTAIM atomic regions (Matta, 2013 ▸), which as we have discussed have been widely used in crystallography (Gatti & Macchi, 2012 ▸). In this regard, we take for granted in the following paragraph that a QTAIM partition is superimposed onto the Born maxima.

**Figure 15:**
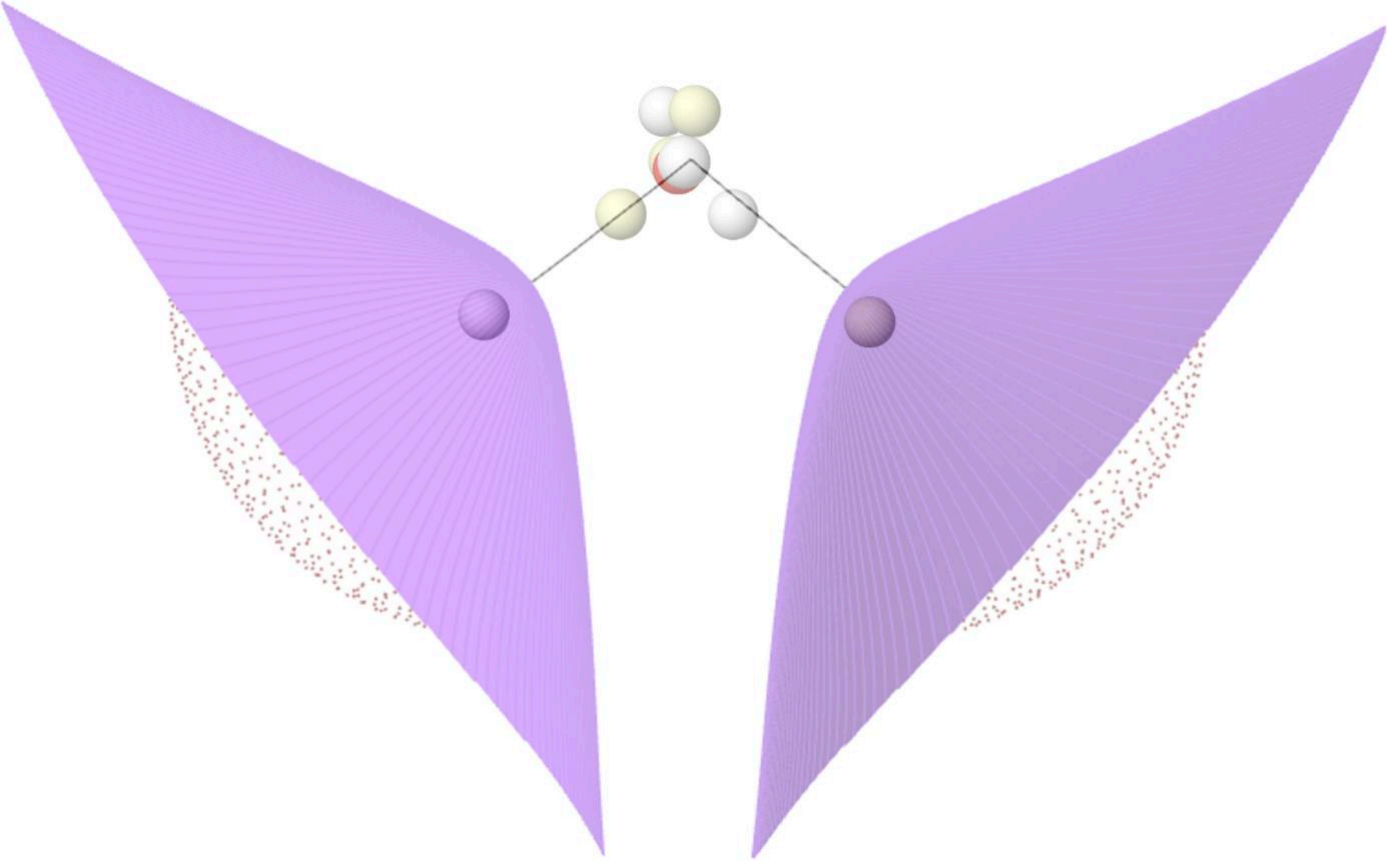
QTAIM interatomic surfaces in H_2_O superimposed onto the HF+J Born maximum of the water molecule.

As an example, a B3LYP/cc-pVDZ calculation in the water molecule (in order to include approximately the effects of electron correlation) renders a QTAIM charge for the O atom of −1.173*e*. Its QTAIM interatomic surfaces are superimposed onto the HF+J maximum, already discussed, in Fig. 15 ▸. As can be seen, the six valence electrons around the oxygen nucleus lie inside the O QTAIM basin. The HF Born maximum of Fig. 7 ▸ (not shown in Fig. 15 ▸) would obviously contain all eight valence electrons in the oxygen basin, as would the correlated mono- or di-ionic maxima. In the latter case, the eight electrons around the O atom would adopt a (distorted) cubic distribution. The QTAIM analysis of ρ provides a very relevant average picture of the electron distribution but is unable to resolve the many subtleties that the Born picture does.

A closer link between the approach described here and QTAIM can be obtained through the analysis of the probability distribution of the basin populations (the so-called electron distribution functions, EDFs) (Martín Pendás *et al.*, 2007*b* ▸). In the same B3LYP calculation, the probability that all ten electrons lie within the oxygen’s QTAIM basin is *p*(0, 10, 0) = 0.40 (the three integers represent the populations of the H1, O and H2 atoms, respectively). In turn, *p*(1, 9, 0) = *p*(0, 0, 1) = 0.21 and *p*(1, 8, 1) = 0.10. These figures can also be obtained by counting the number of electrons in each region during the VMC walks and averaging afterwards. We have already shown (Martín Pendás *et al.*, 2007*a* ▸) that the transferability of the QTAIM atomic expectation values is associated with the constancy of the full EDF. Our current results take this several steps further. Altogether, the persistence of the global and local Born maxima around an atom in the molecule in chemically similar environments causes the constancy of the full electron distribution and, finally, of the electron density.

Finally, a word on the relationship between Born maxima and the VSEPR model (Gillespie & Hargittai, 2012 ▸). Historically, it was Ron Gillespie who convinced Richard Bader (both at McMaster University in Canada) to look at the electron density in search of a theoretical basis for his VSEPR model. He soon found it in ∇^2^ρ = −*L*, whose minima reproduced the basic features of the model very well. Over the years, the role played by the Laplacian was superseded by other scalar fields such as ELF (Silvi & Savin, 1994 ▸). However, as we have recently shown (Menéndez-Herrero *et al.*, 2022 ▸), it is the maxima of *L* that best correlate with the positions of the Born maxima in simple molecules of light elements. An important finding, however, is that these *L* maxima sometimes correspond to one electron (typically in bonded valence shell charge concentrations) and sometimes to two (as in non-bonded valence shell charge concentrations, normally LPs). In the latter, electron correlation will split the *L* maximum into its two constituting electrons, as shown in water in Fig. 7 ▸. In general, with these precautions, the overall Born maxima are expected to be in almost one-to-one correspondence with the *L* maxima, hence in agreement with VSEPR most of the time or with LOCCs at others. Interestingly, Born maxima may also shed light on the occurrence of stereochemically inactive LPs that violate VSEPR rules, but this will be the subject of future work, currently in preparation.

## 7. Conclusions

We have shown in this article how consideration of the multidimensional global and local maxima of the square of the wavefunction, the so-called Born maxima, vividly supports the idea that atoms persist to a large extent in molecules. Although the rules of quantum mechanics forbid strict separability of interacting subsystems, many experimental facts have shown over the years that chemical properties of functional groups are to a large extent transferable. After all, it is this constancy that underlies the science of chemistry.

Born maxima in atoms can be constructed with simple heuristic semiclassical rules that are easy to learn and teach. First, for each atomic shell, we place two subsets of electrons of equal spin on the surface of a sphere and independently minimize their mutual Pauli repulsion. This results in two interpenetrated polyhedra (or polytopes in the general case). In a second step, the two polyhedra are rigidly rotated relative to each other to minimize their Coulombic repulsion. When a principal quantum number shell is filled, the next electrons start a new one at a larger distance from the nucleus, and periodicity sets in. In early transition metal atoms, the M shell can adopt non-perfectly symmetric geometries, leaving a dipole moment and justifying a non-symmetric distribution of N-shell electrons. We have also shown how the Born maxima in excited states typically show different spatial electron distributions.

When a molecule is formed, its Born maximum tends to show clearly identifiable atomic regions that may be neutral or ionic in character. In addition to the global Born maximum, other local maxima are often of great interest. The arrangement of electrons around each atom can always be mapped to that found for the atom in isolation. In some cases, the atom in the molecule corresponds to the ground state of the parent atom, but in many others it is an excited state that corresponds to the atom in the molecule, supporting the concept of atomic promotion. In each case, the spin-coupling rules are easily read from the Born maxima and an exquisitely structured pattern of spin alternation is revealed.

Since the subject is beyond the scope of a single article, we have examined a number of selected examples that are representative of several interesting situations. Thus, we have shown how boron or carbon atoms maintain their ground states in their homodiatomics, while the oxygen atom in water molecules is best described as a mixture of its two intraconfigurational singlets. Similarly, the carbon atoms in triplet and singlet methylidene can be mapped to the isolated ^3^P and ^1^D states of the free C atom.

The information obtained from Born maxima also provides invaluable information about the electron distribution in conflicting cases. For example, the global maximum of ClF_5_ finds a clear LP along the axis of the square pyramid while avoiding any hypervalency due to the ionic configurations of the F moieties. The role of secondary Born maxima was also investigated. The bent geometry of CaH_2_ has been related to the dipole moment of local maxima, which necessarily implies an occupation of the *d* subshell of Ca. This is directly imaged in real space, showing how these techniques can become predictive if used appropriately. We feel this is a very important result of this kind of analysis.

We believe that the analysis of Born maxima in molecules should be generalized as soon as possible to solids, where they would certainly offer new perspectives and open new ways of thinking beyond the fishbowl. Born maxima allow us to draw chemical pictures rigorously based on physical principles and may provide the long-sought predictive character that so many real-space techniques lack. Born maxima cannot be obtained from the electron density, but a more thorough understanding of their structure will certainly help in understanding the scars left on their one-particle projection, now so easily obtained from experiment.

## 8. Related literature

The following references are only cited in the supporting information for this article: Hastings (1970 ▸), Liu & Nocedal (1989 ▸), Lüchow *et al.* (2015 ▸), Metropolis *et al.* (1953 ▸), Pritchard *et al.* (2019 ▸) and Schmidt & Moskowitz (1990 ▸). 

## Supplementary Material

Methodological details, Jastrow factors and electron coordinates at the Born maxima. DOI: 10.1107/S2052252524000915/zx5029sup1.pdf
