# Supplementary material for: Persistence of atoms in molecules: there is room beyond electron densities
Source: IUCrJ. 2024 Feb 20;11(Pt 2):210–23. doi: 10.1107/S2052252524000915 (PMC10916289; doi:10.1107/S2052252524000915)
Supplement: Supplementary file 1 [file m-11-00210-sup1.pdf]

IUCrJ

Volume 11 (2024)

Supporting information for article:

Persistence of atoms in molecules: there is room beyond electron densities

María Menéndez-Herrero and Ángel Martín Pendás

Supporting Information

Persistence of atoms in molecules:
there is room beyond electron densities

María Menéndez Herrero, and Ángel Martín Pendás*

Departamento de Química Física y Analítica. Facultad de Química. Universidad de Oviedo. 33006 Oviedo. Spain.

E-mail: ampendas@uniovi.es

Contents

1 Methodological details **2**

1.1 Electronic structure calculations, ELF and LOL. 2

1.2 The Slater-Jastrow wavefunction 2

1.3 Optimization of the wavefunction with *Amolqc* 3

1.4 Search for maxima of $|\Psi|^2$ 4

1.5 Ground state sm21 Jastrow factors 5

1.6 Ground state de444 Jastrow factors 7

2 Electron coordinates at the HF+J Born maximum **12**

1 Methodological details

1.1 Electronic structure calculations, ELF and LOL.

Given that the focus in this paper is to demostrate evidence that the electronic structure of atoms persists in molecules to a much greater extent than it has been usually admitted using Born maxima, we have chosen as simple level of theory as possible, and all calculations have been performed at the single-determinant or subshell active space level, with the cc-pVDZ basis set. Basis sets have been retrieved from the EMSL server (Pritchard *et al.*, 2019). Wavefunctions for the ground states of atoms and molecules have been obtained through the GAMESS suite (Schmidt *et al.*, 1993). The PROMOLDEN code (Martín Pendás and Francisco, 2023) has also been used to provide some electron densities using the very same wavefunctions.

1.2 The Slater-Jastrow wavefunction

An efficient and compact form of including electron correlation effects in Quantum Monte Carlo (QM) calculations is by multiplying a Slater determinant or a linear combination of Slater determinants Φ by a Jastrow factor e^U according to Eq. 1

$$\Psi = \Phi e^U. \tag{1}$$

$\Phi = \Phi(\mathbf{r}_1, \ldots, \mathbf{r}_N)$ and U are respectively antisymmetric and symmetric functions of the cartesian coordinates of the N electrons of the system, $\mathbf{r}_1, \ldots, \mathbf{r}_N$. U is usually expressed as a linear combination of many-body terms. Many different forms of the Jastrow factor have been used in the literature. Here we use either the Schmidt and Moskowitz (sm) ansatz, (Schmidt and Moskowitz, 1990) or the generalized double exponential (de) one defined by Lüchow and coworkers. (Lüchow *et al.*, 2015) In the first case,

$$U = \sum_{A,i<j} U_{Aij}, \tag{2}$$

where the symbol A stands for nuclei, and i and j represent electrons. Each term U_{Aij} of Eq. 2 has the expression

$$U_{Aij} = \sum_{k}^{N(A)} \Delta(m_{kA}, n_{kA}) c_{kA} \left(\bar{r}_{iA}^{m_{kA}} \bar{r}_{jA}^{n_{kA}} + \bar{r}_{jA}^{m_{kA}} \bar{r}_{iA}^{n_{kA}} \right) \bar{r}_{ij}^{o_{kA}}. \tag{3}$$

The k sum runs over the $N(A)$ terms used to express the correlation function of atom A, where the c_{kA} are variational parameters that are optimized and m_{kA}, n_{kA} and o_{kA} are

predefined integers. The $\bar{r}$ functions represent scaled nuclear-electron and electron-electron distances, described by Eqs. 4 and 5, respectively

$$\bar{r}_{iA} = \frac{a_A r_{iA}}{1 + a_A r_{iA}}, \tag{4}$$

$$\bar{r}_{ij} = \frac{b_A r_{ij}}{1 + b_A r_{ij}}, \tag{5}$$

where r_{iA} is the distance between electron i and nucleus A, and r_{ij} the distance between electrons i and j. The parameters m_{kA}, n_{kA} and o_{kA} are integers, and the function Δ is one for $m \neq n$ and $\frac{1}{2}$ for $m = n$. Finally, a_A and b_A are optimizable parameters which in this work are taken as $a_A = b_A = 1$.

In the double exponential ansatz, $U = U_{ee} + U_{en} + U_{een}$. U_{ee} and U_{en} are written as power series of scaled nuclear-electron and electron-electron distances ($U = \sum_k c_k \bar{r}^k$) with fixed linear terms because the orbitals employed satisfy at least approximately the cusp conditions, and optimizable parameters starting at $k = 2$. The U_{een} term is written as a power series of scaled $ee(r_{ij})$, and $en(r_i)$ coordinates symmetric with respect to particle exchange. In this work we have used a de444 ansatz terminating the series at the quartic power $k = 4$ for both the ee, en, and een terms. This means that in de444 calculations we need to specify 3 parameters for the U_{ee} term, 3 parameters for each type of nucleus to build the U_{en} contribution, and 7 U_{een} parameters per nuclear type, since the number homogeneous symmetric three-body polynomials of degree 4 is 7, as found in Table S1.

We report here the optimized parameters of all the systems studied. The notation is direct in the case of sm ansatzes. In order to save space, we directly write the coefficients in the de444 cases: A first line that contains the 3 U_{ee} coefficients is followed by as many lines as different nuclear types with their corresponding 3 U_{en} coefficients. Finally, we write seven lines with as many columns as the number of nuclear types with the U_{een} coefficients ordered as in Table S1.

1.3 Optimization of the wavefunction with *Amolqc*

In this work, a Slater-Jastrow wavefunction ansatz has been used according to Eq. 1 in variational Monte Carlo (VQMC) calculations. The prefactor Φ was obtained at the HF/cc-pVDZ level of theory using the GAMESS program (Schmidt *et al.*, 1993). This is a single Slater determinant in the case of ^1S atomic states or closed systems, and a symmetry-adapted intra-shell complete active space (CAS) linear combination of Slater determinants in the case of open-shell atoms. Thus, the non-Jastrow VQMC exact energy

Table S1: Three-body polynomials of degree p=4

p	n	
4	7	$r_i^3 r_j + r_i r_j^3$
		$r_i^2 r_j^2$
		$r_{ij}(r_i^3 + r_j^3)$
		$r_{ij}(r_i^2 r_j + r_i r_j^2)$
		$r_{ij}^2(r_i^2 + r_j^2)$
		$r_{ij}^2 r_i r_j$
		$r_{ij}^3(r_i + r_j)$

should replicate that of the standard electronic structure code if no other correction (e.g. a cusp correction) is applied.

The optimization of the Jastrow factors was performed through the *Amolqc* QMC code (Lüchow *et al.*, 2021). In a first step, samples of 1500 walkers were used, after which an optimization with respect to the variance of the energy was carried out. A new sample of 1500 walkers is then generated using as trial wavefunction the one containing the new parameters, which is followed by another optimization, this time with respect to the energy. The reference energies used in each case were those obtained from the HF/cc-pVDZ calculation for each system.

1.4 Search for maxima of $|\Psi|^2$

Instead of proceeding with the search of the maxima of $|\Psi|^2$, it is more efficient (and equivalent) to perform the minimization of the function $-\ln(|\Psi|^2)$. Both this minimization and the sampling of $|\Psi|^2$ to generate the $\{\mathbf{r}_i\}$ $(i = 1, \ldots, N)$ electronic distributions are also performed with the *Amolqc* program (Lüchow *et al.*, 2021). The variational Monte Carlo (VMC) method is used to sample $|\Psi|^2$ in the regions where it is large via the Metropolis-Hastings algorithm (Metropolis *et al.*, 1953; Hastings, 1970). Then, the algorithms to perform a local minimization of $-\ln(|\Psi|^2)$ are invoked. To reduce the computational cost of these local minimizations, a combination of two methods is used. First, the steepest descent (SD) algorithm is applied and, after five steps, the code is switched to the L-BFGS algorithm (Liu and Nocedal, 1989) to avoid the well known size-step problem of SD.

1.5 Ground state sm21 Jastrow factors

Table S2: Values of the Jastrow factors using the sm21 ansatz.

m	n	o	He (^{1}S)	Li (^{2}S)	Be (^{1}S)	B (^{2}P)	C (^{3}P)	N (^{4}S)	O (^{3}P)	F (^{2}P)	Ne (^{1}S)
0	0	1	0.50000	0.50000	0.50000	0.50000	0.50000	0.50000	0.50000	0.50000	0.50000
0	0	2	0.04515	-0.11837	0.35626	0.22519	-0.30163	-0.22696	-0.28757	-0.57618	-0.46260
0	0	3	0.91353	1.25185	-1.12013	-1.15077	0.17182	-0.19125	0.07705	0.35987	0.63859
0	0	4	-0.58355	-1.22020	1.10267	1.27545	-0.14770	0.47955	0.37095	-0.04002	-0.22152
2	0	0	1.29346	0.12504	0.15277	0.04201	0.01286	-0.00860	-0.03426	-0.00516	-0.03356
3	0	0	-3.69939	-0.91186	-1.27497	-0.58707	-0.26299	-0.03338	0.17048	-0.06721	0.23202
4	0	0	4.24143	1.61450	3.21442	1.66338	0.62255	-0.17295	-0.93600	-0.13308	-1.52305
2	2	2	-2.10484	-1.44821	-3.22536	-2.20962	-1.00095	-0.55128	0.04675	-0.36272	0.83571

m	n	o	Na (^{2}S)	Mg (^{1}S)	Al (^{2}P)	Si (^{3}P)	P (^{4}S)	S (^{3}P)	Cl (^{2}P)	Ar (^{1}S)	Ca (^{1}S)	Sc (^{2}D)	Mn (^{6}S)
0	0	1	0.50000	0.50000	0.50000	0.50000	0.50000	0.50000	0.50000	0.50000	0.50000	0.50000	0.50000
0	0	2	-0.93833	-0.90504	-0.88816	-1.05995	-0.85870	-0.88860	-0.84474	-0.97043	-1.39895	-1.39895	-1.81959
0	0	3	2.04824	1.76097	1.58601	1.72469	1.26859	1.25958	1.02858	1.41428	2.28671	2.28671	3.33578
0	0	4	-1.42415	-1.09497	-0.92255	-1.01532	-0.55573	-0.55076	-0.27496	-0.62367	-1.33081	-1.33081	-2.16167
2	0	0	0.00215	0.00626	0.01198	0.00390	0.01932	0.01402	0.01396	0.00880	-0.00029	-0.00029	0.00083
3	0	0	-0.13193	-0.21382	-0.29575	-0.21716	-0.38414	-0.35450	-0.37649	-0.32350	-0.17492	-0.17492	-0.18066
4	0	0	-0.35748	-0.03486	0.28924	0.22199	0.65408	0.63099	0.74921	0.59231	0.26082	0.26082	0.43477
2	2	2	0.32010	0.00008	-0.32497	-0.22356	-0.72406	-0.70665	-0.87442	-0.71179	-0.21528	-0.21528	-0.39070

m	n	o	Kr (^{1}S)	KCl	ClF_4
0	0	1	0.50000	0.50000	0.50000
0	0	2	-1.37662	-1.44054	-0.48926
0	0	3	2.37461	2.29062	-0.41930
0	0	4	-1.40297	-1.28943	0.94979
2	0	0	-0.00390	-0.00179	-0.00042
3	0	0	-0.23582	-0.07857	-0.06066
4	0	0	0.36419	0.08812	0.08402
2	2	0	-0.35688	-0.00962	-0.14379
2	0	2		-0.00406	-0.00135
2	2	2		-0.02233	-0.00796
2	2	2		-0.07604	-0.01957
2	2	2		0.11813	-0.03499

1.6 Ground state de444 Jastrow factors

Table S6: Values of the Jastrow factors using the de444 ansatz.

	C ^{3}P			C ^{1}D		
U_{ee}	-0.3854960D+00	0.1755614D+00	0.3585114D+00	-0.6077853D-01	-0.1983510D+00	-0.4047527D+00
U_{en}	0.3452847D+01	0.6478277D+00	0.7033315D-01	-0.1746662D+01	0.7177462D+01	-0.5266420D+01
U_{een}	-0.2224873D+01			0.2224912D+00		
	0.2225190D+01			0.3907403D+00		
	0.3048221D+00			-0.4460639D+00		
	0.1595167D+01			0.1278566D+01		
	0.4028475D+00			-0.1788419D+01		
	0.9178739D+00			0.2203232D+00		
	-0.8670781D+00			0.2166374D+00		

	C ^{1}S			C ^{5}S		
U_{ee}	-0.3816557D-01	0.2209412D+00	-0.7047029D+00	-0.4332929D-01	-0.5268941D-01	0.1164651D+01
U_{en}	-0.1879107D+01	0.6836075D+01	-0.3897355D+01	-0.1630536D+01	0.8202279D+01	-0.2281187D+01
U_{een}	0.4956124D+00			0.1431664D-01		
	0.4341210D+00			0.7518016D+00		
	-0.1666294D+01			0.2016863D+00		
	0.2529022D+01			0.6151526D+00		
	-0.9889772D+00			-0.2649638D+01		
	0.1158267D+01			0.2138510D+00		
	-0.7608627D-01			-0.1424760D+01		

	N ^{4}S			N ^{2}D		
U_{ee}	-0.4299259D+00	-0.1475833D+00	0.1112110D+01	-0.3773498D+00	-0.1949969D+00	0.1846422D+00
U_{en}	-0.2200585D+01	0.6270702D+01	-0.2358592D+01	-0.2502122D+01	0.7017191D+01	-0.3622213D+01
U_{een}	0.1973060D+00			0.3111847D+00		
	-0.4001552D+00			-0.3915555D+00		
	0.7655112D+00			-0.1083052D+00		
	-0.5437038D+00			0.3777211D+00		
	-0.2995394D+01			-0.2242788D+01		
	-0.6116118D+00			0.1767854D+00		
	-0.1549692D+01			-0.4847547D+00		

	N ^{2}P			O ^{3}P		
U_{ee}	-0.3259778D+00	-0.3321595D-01	-0.8284658D-02	-0.5834902D+00	0.9344574D-02	0.6939061D+00
U_{en}	-0.2118587D+01	0.6789379D+01	-0.3100372D+01	-0.2853313D+01	0.6947474D+01	-0.2460345D+01
U_{een}	0.3105302D+00			0.3626365D+00		
	-0.1527095D+00			-0.7693951D+00		
	-0.6172003D+00			0.1181771D+00		
	0.1004547D+01			-0.1831652D+00		
	-0.1802405D+01			-0.2565198D+01		
	0.5543049D+00			-0.8630813D-01		
	-0.4783698D+00			-0.1359789D+01		

	O ^{1}D			O ^{1}S		
U_{ee}	-0.5722252D+00	0.3189451D+00	0.1484574D+00	-0.4846866D+00	0.2096465D-01	0.1943036D+00
U_{en}	-0.7813577D+00	0.6716283D+01	-0.3936079D+01	-0.2834870D+01	0.7747148D+01	-0.2449673D+01
U_{een}	-0.3408245D+00			0.4787532D+00		
	0.3410606D+00			-0.6057736D+00		
	-0.2792814D+00			-0.1151487D+01		
	0.1070466D+01			0.1178707D+01		
	-0.1437952D+01			-0.1618170D+01		
	0.4715227D+00			0.1014411D+01		
	-0.9330904D+00			-0.9310294D+00		

	B_2			C_2		
U_{ee}	-0.1211072D+01	-0.4785820D+00	0.9720364D+00	-0.9726496D+00	-0.4924554D+01	-0.2899567D+00
U_{en}	0.5492911D+01	-0.4355220D-01	0.1924215D+00	-0.3762510D+01	0.4126299D+01	-0.1797853D+01
U_{een}	-0.1948925D+01			-0.1492422D+00		
	0.8835331D+00			-0.2265616D+01		
	-0.2075294D+00			-0.3622317D+01		
	0.7937561D+00			0.2119401D+01		
	0.1538933D+01			0.3671828D+01		
	0.1576244D+01			0.4441172D+01		
	-0.3841996D+00			0.1908187D+01		

	H_2O			$^3B_1$ CH_2		
U_{ee}	0.5628498D+00	-0.1863159D+01	0.1876630D+00	-0.1812467D+01	-0.5583265D+00	0.3012769D+00
U_{en}	-0.4286898D+01	0.1141623D+02	-0.4002033D+01	-0.1007112D+01	0.4933132D+01	-0.3354782D+01
	0.3559768D+01	-0.2435084D+00	-0.3435970D+00	-0.8590858D+00	0.3848798D+01	-0.7848064D+00
U_{een}	0.5160307D+00	-0.8785756D+00		-0.2283113D+00	-0.7648497D+00	
	-0.7454647D+00	0.1084758D+01		0.1035025D-01	-0.4628388D+00	
	-0.6619671D+00	-0.1317746D+00		0.1004569D+00	-0.4352948D+00	
	0.5385821D+00	0.7390330D+00		0.3250435D+00	0.2431389D+00	
	-0.1947696D+01	0.7724228D+00		-0.1079270D+01	0.8884036D+00	
	0.6537757D+00	0.6324898D+00		0.6558507D-01	0.1490660D+01	
	-0.7244500D+00	0.7410970D+00		-0.1722540D+00	0.2708729D-03	

	$^1A_1$ CH_2			C_3O_2		
U_{ee}	-0.3140368D+01	-0.1583158D+00	-0.4459179D+00	-0.4307061D+01	-0.1213401D+01	0.1069737D+01
U_{en}	0.1955658D+01	0.5305394D+01	-0.5729714D+01	0.1188667D+02	-0.6000688D+01	0.2456686D+01
	0.4955691D+01	-0.7493547D+00	-0.1764683D+01	-0.6939132D+02	0.7493113D+02	0.3378821D+01
U_{een}	-0.1065777D+01	-0.2245531D+01		-0.3037326D+01	0.2966614D+00	
	0.1457604D+01	-0.1094280D+00		0.1879806D+01	-0.1590461D+01	
	-0.8735850D+00	-0.9615546D+00		0.9366316D+00	-0.1177895D+01	
	0.2383411D+01	0.8720930D+00		0.9549782D+00	0.1069030D+01	
	0.4228297D+00	0.3034236D+01		0.1804379D+01	0.2437950D+00	
	0.1006324D+01	0.2433875D+01		0.1653133D+01	0.3327910D+01	
	0.2345976D+00	0.3312423D-01		0.1108192D+00	-0.2012562D+00	

	CaH_2			MgH_2		
U_{ee}	0.3085923D+00	0.1892659D+01	0.2353929D+00	-0.6994755D+01	0.2028566D+01	-0.7296799D+00
U_{en}	-0.1940124D+02	0.1913086D+02	-0.8165175D+00	0.8817573D+00	0.2570485D+01	0.2650024D+01
	0.5119001D+01	0.1451507D+01	-0.9934973D+01	-0.2401364D+02	0.2803423D+02	-0.7101009D+01
U_{een}	0.6758268D+00	-0.1405145D+01		-0.7192811D+00	-0.8636083D+00	
	0.7203649D+00	0.3523238D+00		0.1410856D+00	-0.2928427D+01	
	-0.9684048D+00	0.1378785D+00		-0.9282648D+00	0.4543198D+00	
	0.1664802D+01	0.8328029D+00		0.1152609D+01	-0.1531628D+01	
	0.2016651D+00	0.4192780D+00		0.8046316D+00	0.2392510D+00	
	0.1201069D+01	0.1000826D+00		0.1520770D+01	0.1656927D+01	
	-0.2915276D+00	-0.1784818D+01		-0.2078133D-01	-0.2791755D+00	

2 Electron coordinates at the HF+J Born maximum

Nuclei at the origin of the reference System. Majority spin electrons followed by minority spin ones. All data in au.

Li

```
0.000000     0.000000     0.000000
0.421021     0.465077    -1.604987
0.000000     0.000000     0.000000
```

Be

```
0.000000     0.000000     0.000000
-1.040667     0.317501    -0.275763
1.059810    -0.263955    -0.258784
0.000000     0.000000     0.000000
```

B

```
0.000007    -0.000006    -0.817714
0.000001    -0.000004     0.817680
0.000000     0.000000     0.000000
0.841612     0.000154     0.000075
0.000000     0.000000     0.000000
```

C

```
0.550702     0.000897    -0.285043
0.000000     0.000000     0.000000
-0.028222    -0.000045     0.619002
-0.522495    -0.000852    -0.333868
0.000000     0.000000     0.000000
-0.547411    -0.394533    -0.000032
```

N

```
0.452959     0.102266    -0.162067
```

-0.284045	-0.131542	-0.379356
0.021393	-0.368988	0.324478
0.000000	0.000000	0.000000
-0.190307	0.398264	0.216945
0.000000	0.000000	0.000000
-0.512166	-0.031832	0.228844

O

0.000000	0.000000	0.000000
0.122054	0.219704	-0.315277
0.124408	0.174560	0.341495
-0.407731	-0.024291	-0.000181
0.156534	-0.370646	-0.026044
0.442312	0.001004	-0.000001
0.000000	0.000000	0.000000
-0.442312	-0.001004	-0.000000

F

0.000000	0.000000	0.000000
-0.011966	0.331751	0.103234
-0.278540	-0.169014	0.120292
0.288161	-0.149481	0.123338
0.002313	-0.013118	-0.344136
0.166533	0.320455	-0.000000
-0.360440	-0.015811	0.000000
0.000000	0.000000	0.000000
0.193880	-0.304648	-0.000000

Ne

0.290334	0.089594	-0.022201
-0.049682	-0.115557	0.277475
-0.056950	-0.212889	-0.210355
-0.183702	0.238851	-0.044919

0.000000	0.000000	0.000000
-0.100984	0.021407	-0.286632
-0.100738	-0.259376	0.124056
0.000000	0.000000	0.000000
-0.102928	0.236677	0.161875
0.304650	0.001291	0.000701

Na

0.919778	0.264971	1.834583
0.218399	-0.145186	0.011813
-0.116465	-0.036245	-0.231858
0.061809	0.253578	0.028129
-0.163573	-0.072098	0.192255
0.000000	0.000000	0.000000
0.110869	-0.177160	0.158491
0.000000	0.000000	0.000000
0.121333	-0.002647	-0.232527
-0.253501	-0.058710	-0.032986
0.021298	0.238517	0.107021

Mg

0.121635	-0.103864	0.175085
0.015915	0.235654	0.021566
0.089911	-0.066568	-0.209112
1.564740	0.507609	-0.104216
-0.226986	-0.065070	0.012430
0.000000	0.000000	0.000000
0.602824	-1.346749	-0.734723
-0.090211	0.197396	0.093853
-0.066529	-0.017096	-0.227019
-0.079766	-0.188211	0.120209
0.000000	0.000000	0.000000

0.236687	0.007504	0.012736

Al

-0.000002	0.000005	-1.601623
-0.037819	0.201314	-0.069832
-0.000004	0.000007	1.601611
0.191749	-0.067387	-0.074291
-0.155746	-0.131876	-0.071993
0.000000	0.000000	0.000000
0.001805	-0.002039	0.215458
1.388305	-0.000232	-0.000133
0.069262	-0.063878	0.195162
0.076242	-0.134917	-0.151518
0.070972	0.200881	-0.039727
0.000000	0.000000	0.000000
-0.215992	-0.002085	-0.003916

Si

-1.222127	-0.004374	0.696204
1.215462	0.004339	0.707789
0.000000	0.000000	0.000000
0.000636	-0.198657	-0.000929
0.178018	0.066805	-0.053980
-0.042307	0.064997	0.181801
0.006707	0.000019	-1.406362
-0.136344	0.066142	-0.126898
-0.162505	-0.112912	0.009832
0.063019	0.009046	-0.188320
0.142510	-0.079014	0.113921
-0.042633	0.183166	0.064568
0.981793	0.707710	0.000249
0.000000	0.000000	0.000000

P

-0.900136	-0.834106	-0.051544
0.181433	0.334942	1.167701
0.000000	0.000000	0.000000
0.121442	0.113125	-0.075454
-0.315216	0.984090	-0.663951
-0.115472	-0.059351	-0.127994
1.033921	-0.484928	-0.452207
0.087066	-0.142111	0.073911
-0.093036	0.088336	0.129537
-0.561304	-0.908175	-0.132059
-0.162649	0.039448	-0.075696
-0.026158	-0.080047	0.163245
0.095764	0.154275	0.022450
0.000000	0.000000	0.000000
0.092778	-0.114104	-0.110062

S

-0.032748	1.067088	-0.004065
-0.066398	0.154661	-0.010311
0.512921	-0.342288	-0.862074
-0.988873	-0.381218	-0.003184
0.010095	-0.067224	-0.154272
0.504898	-0.335911	0.869258
0.000000	0.000000	0.000000
0.154244	0.009438	0.067385
-0.097954	-0.096848	0.097197
-0.000490	0.168286	0.000532
0.062172	-0.056732	0.147494
0.097053	-0.055763	-0.127715
-0.005173	1.028913	-0.000001
0.005163	-1.028947	-0.000001

-0.158731	-0.056850	-0.020318
0.000000	0.000000	0.000000

Cl

0.006245	0.139135	0.071846
0.000000	0.000000	0.000000
0.172873	-0.878089	0.300156
-0.013045	-0.113592	0.107169
0.003906	0.017408	-0.939410
0.666018	0.582194	0.328726
0.130895	-0.021648	-0.083397
-0.843846	0.279758	0.316504
-0.124094	-0.003893	-0.095611
0.000488	-0.000009	-0.158292
0.423117	-0.835677	-0.000010
0.136630	0.055899	0.052836
-0.020151	-0.146405	0.052362
0.000000	0.000000	0.000000
-0.935050	0.050323	-0.000010
0.509932	0.785674	-0.000010
-0.116959	0.090515	0.052047

Ar

-0.705269	0.454947	0.149410
0.446588	0.365853	-0.627228
-0.093518	-0.010466	0.112357
0.133467	0.033431	0.050479
0.000000	0.000000	0.000000
-0.216084	-0.792013	-0.229623
0.005343	-0.127991	-0.071198
-0.045292	0.105025	-0.091639
0.474765	-0.028787	0.707442
-0.074817	-0.001691	-0.126012

0.000000	0.000000	0.000000
0.631119	0.246250	0.517450
0.041187	-0.136432	0.034196
0.230960	-0.709575	-0.412146
-0.161136	0.617323	-0.565378
-0.085116	0.053511	0.106636
0.118746	0.084612	-0.014820
-0.700943	-0.153997	0.460074

K

-0.074786	-0.069978	0.090482
0.681845	-0.253500	0.005088
0.092813	0.075091	0.066060
0.003365	0.707855	0.163714
0.063296	-0.087123	-0.083796
1.461582	1.574323	-1.887796
-0.081405	0.081928	-0.072647
-0.289112	-0.094864	-0.660374
-0.402505	-0.366289	0.499821
0.000000	0.000000	0.000000
-0.057843	-0.074021	-0.099065
-0.006539	-0.593097	0.427923
0.029964	0.124362	-0.047702
-0.666447	0.301135	-0.009479
0.000000	0.000000	0.000000
0.190555	-0.191790	-0.679580
0.482420	0.483736	0.261141
0.114488	-0.061502	0.041823
-0.086609	0.011162	0.104945

Ca

-0.609942	0.262774	0.158904
-0.116839	0.054281	0.019370

0.000000	0.000000	0.000000
2.147029	-0.883466	-0.529224
-0.005431	-0.126414	0.029833
0.333569	0.430233	-0.387565
0.347244	-0.111005	0.560020
0.088088	0.058578	0.075553
0.034006	0.013635	-0.124725
-0.084467	-0.576379	-0.327986
-0.587708	-0.142796	0.284699
0.000000	0.000000	0.000000
0.399228	-1.242187	1.991961
-0.098103	-0.082696	0.020893
-0.022907	0.068018	-0.108730
-0.105807	0.348415	-0.577702
0.110808	-0.066695	-0.013112
0.366732	0.369743	0.418974
0.010166	0.081476	0.100785
0.324272	-0.567507	-0.138612

Mn

-0.145604	-0.375060	0.217551
0.067011	0.065977	0.038664
-0.078930	-0.006585	0.060808
-0.031040	0.027823	-0.092740
1.281887	-1.045483	-0.652985
0.334278	-0.021041	-0.243322
-0.069753	0.091193	0.442552
0.305924	0.353456	0.101934
0.342985	-0.198843	0.216856
0.041730	-0.090300	-0.004919
-0.201080	0.359322	0.045708
-0.448944	-0.056364	-0.065623
-0.087687	0.200288	-0.425567

-0.034446	-0.345715	-0.288961
0.000000	0.000000	0.000000
-0.286461	-0.238394	-0.326661
1.035354	0.878326	1.176154
-0.004681	0.100228	-0.007654
0.093366	-0.030924	-0.021293
-0.023894	-0.027612	0.093772
0.000000	0.000000	0.000000
-0.064870	-0.041745	-0.064906
-0.014909	0.479317	-0.089942
0.459644	-0.158814	-0.040353
-0.162609	-0.085781	0.452029

Kr

-0.808472	-0.237608	-0.578317
0.506180	-0.850743	0.254076
0.002564	0.165320	-0.217332
-0.031452	0.056351	-0.015411
0.045693	0.008419	0.269095
0.133914	-0.189333	0.071080
-0.041918	-0.039881	0.036826
0.061293	-0.172440	-0.223822
0.051951	0.017452	0.037399
-0.216474	0.130693	0.103098
0.000000	0.000000	0.000000
-0.196312	-0.035317	-0.138011
0.070379	0.253386	0.075492
-0.317742	0.475472	0.852061
-0.167114	-0.208548	0.110313
0.623184	0.624214	-0.524443
0.264731	0.043046	-0.051335
0.022597	-0.029682	-0.057551
-0.215595	0.116071	-0.153737

-0.027291	-0.115756	-0.211396
-0.792001	-0.273293	0.592526
-0.002190	-0.065987	0.006562
0.018256	-0.142671	0.232741
0.011105	0.242247	0.005049
0.215090	0.084747	0.145344
0.037222	1.019014	-0.068996
-0.202572	-0.182572	0.014239
-0.115637	-0.406210	-0.930676
0.871232	-0.345893	0.417557
-0.059525	0.022073	-0.025949
0.007508	0.024432	0.061228
0.188145	0.098447	-0.196210
0.054513	0.017095	-0.037946
0.184607	-0.199472	-0.026491
-0.172089	0.101648	0.186075
0.000000	0.000000	0.000000

KCl

4.484135	0.471245	-0.702411
4.601539	0.023180	-0.085069
4.479778	0.504354	0.679291
4.347906	0.031654	-0.085762
3.777681	-0.471536	0.008846
4.468711	-0.148134	0.046530
-0.549609	-0.620246	-0.822113
-0.579798	0.040640	-0.150049
0.223048	0.629245	-0.018488
4.476567	0.093207	0.124171
4.474191	0.000000	0.000000
-1.499589	0.556358	-0.015275
-0.832875	0.089156	-0.002481
-0.579680	0.049127	0.147412

5.153294	-0.504519	0.013976
-0.706546	-0.179189	0.005125
-0.675842	0.000000	0.000000
-0.548468	-0.572337	0.856074
4.345482	-0.023102	0.084908
-1.432901	-0.647352	0.019029
-0.615888	0.573732	-0.857578
4.451966	-0.503940	-0.679189
5.185263	0.458505	-0.008541
4.599117	-0.031709	0.085923
4.479005	0.148119	-0.046523
-0.614740	0.621675	0.823487
4.471123	-0.093213	-0.124176
4.447675	-0.470705	0.702255
4.474191	0.000000	0.000000
-0.516027	-0.090298	0.002510
-0.770042	-0.040191	0.149656
-0.642910	0.179039	-0.005119
-0.675842	0.000000	0.000000
3.809953	0.516623	-0.014237
-0.770165	-0.048654	-0.147043
0.286264	-0.541039	0.014865

ClF_5

-12.914709	-1.331293	-2.702667
-10.416604	0.591276	-2.338404
-10.293045	0.655411	-2.225521
-12.567802	2.604166	-1.127692
-8.268735	-1.733883	-2.986471
-7.698517	2.026028	-1.340239
-8.159156	2.142326	-1.719126
-10.643298	0.911387	-2.991151
-12.293329	2.726213	-0.925871

-7.881178	2.297963	-1.483227
-10.354730	-0.404419	0.637779
-10.513622	0.721474	-2.397531
-10.337303	0.508690	-2.471163
-8.324692	-1.816294	-3.572284
-12.045454	2.431681	-0.883175
-8.051479	2.481286	-1.226525
-8.734723	-2.023688	-3.195365
-12.790997	-1.405117	-3.035845
-8.497871	-1.762340	-3.259324
-13.125319	-1.037651	-2.722024
-8.701479	-1.422683	-3.278370
-13.089025	-1.610367	-2.562526
-7.665909	2.514112	-1.677610
-12.118738	2.982914	-1.113964
-10.049224	-0.538980	0.125789
-9.912421	-0.798410	0.676713
-10.520918	0.475175	-2.250485
-10.420349	-0.937780	0.402912
-12.606720	-1.234877	-2.488561
-10.184640	-0.678733	0.481011
-12.405963	2.849044	-0.609430
-10.300872	0.877616	-3.034508
-8.247477	-1.513546	-3.339181
-7.673072	2.389873	-1.207271
-10.314003	0.703337	-2.420856
-9.946314	-0.434008	0.590857
-12.293329	2.726213	-0.925871
-8.712777	-1.804868	-3.548728
-7.692961	2.130815	-1.743223
-10.317349	0.456674	-2.274197
-12.483383	2.490336	-0.736125
-10.497696	0.674039	-2.201673

-10.184640	-0.678733	0.481011
-8.044790	2.587375	-1.628926
-13.058717	-1.268187	-2.381685
-12.033638	2.867617	-0.723206
-10.416604	0.591276	-2.338404
-12.116506	2.526620	-1.219193
-12.661835	-1.035593	-2.778793
-12.723252	-1.634272	-2.695080
-8.497871	-1.762340	-3.259324
-10.011941	-0.967369	0.355992
-13.167560	-1.342657	-2.961087
-10.375405	-0.515351	0.163262
-8.349675	-2.072041	-3.159176
-10.419868	-0.761645	0.735012
-8.162096	2.054830	-1.373723
-10.537447	0.526876	-2.447775
-7.881178	2.297963	-1.483227
-12.914709	-1.331293	-2.702667
-12.504771	2.983919	-1.066325
-8.718474	-1.613552	-2.977929

C ^3P

0.000000	0.000000	0.000000
-0.034353	0.703748	-0.147224
-0.592287	-0.381624	-0.147225
0.626640	-0.322123	-0.147225
0.000000	0.000000	0.000000
0.000000	0.000003	0.787302

C ^1D

0.357355	-0.650564	0.131412
-0.357374	0.650554	-0.131400

0.000000	0.000000	0.000000
0.000000	0.000000	0.000000
0.117279	-0.028323	0.744066
-0.117268	0.028322	-0.744083

C ^1S

0.357355	-0.650564	0.131412
-0.357374	0.650554	-0.131400
0.000000	0.000000	0.000000
0.000000	0.000000	0.000000
0.117279	-0.028323	0.744066
-0.117268	0.028322	-0.744083

C ^5S

0.000000	0.000000	0.000000
0.432474	-0.098941	-0.564080
-0.164835	0.696219	0.055840
-0.601078	-0.381701	-0.089552
0.333438	-0.215577	0.597792
0.000000	0.000000	0.000000

N ^4S

0.000000	0.000000	0.000000
0.432474	-0.098941	-0.564080
-0.164835	0.696219	0.055840
-0.601078	-0.381701	-0.089552
0.333438	-0.215577	0.597792
0.000000	0.000000	0.000000

N ^2D

0.000000	0.000000	0.000000

0.000000	-0.243550	0.551743
-0.000000	-0.356049	-0.486791
0.000000	0.599598	-0.064951
0.624407	-0.000000	-0.000000
0.000000	0.000000	0.000000
-0.624407	0.000000	0.000000

N ^2P

0.000000	0.000000	0.000000
0.565847	-0.167031	-0.074060
-0.326819	-0.234523	-0.437899
-0.210668	0.353911	0.451216
0.540331	0.093990	0.297397
-0.600866	0.007707	-0.167739
0.000000	0.000000	0.000000

O ^3P

-0.274330	0.088834	-0.409996
0.274329	-0.409997	-0.088835
0.000000	0.000000	0.000000
0.274330	0.409997	0.088833
-0.274328	-0.088834	0.409997
-0.536659	0.000000	0.000000
0.000000	0.000000	0.000000
0.536659	-0.000000	0.000000

O ^1D

0.242379	-0.332306	-0.318721
0.242366	0.332308	0.318729
0.000000	0.000000	0.000000
-0.513414	-0.000013	0.000001
-0.242371	0.332302	-0.318724
-0.242378	-0.332303	0.318722

0.513418	-0.000007	0.000002
0.000000	0.000000	0.000000

O ^1S

-0.437773	-0.263108	0.085846
0.000000	0.000000	0.000000
0.002794	0.516692	0.035517
0.434978	-0.253584	-0.121363
0.437773	0.263108	-0.085846
-0.434978	0.253585	0.121363
0.000000	0.000000	0.000000
-0.002794	-0.516692	-0.035517

$\mathbf{B}_2$

0.857825	-0.262306	-0.246451
0.000000	0.000000	0.000000
-0.867952	-0.261494	-0.209015
0.000000	0.000000	0.000000
0.014309	0.740111	0.643557

$\mathbf{C}_2$

0.191620	0.622305	-0.824700
0.000000	0.000000	1.197141
-0.374842	0.000001	1.877355
0.000000	0.000000	-1.197141
-0.520638	-0.000080	-1.704170
0.191690	-0.622219	-0.824603
0.520650	-0.000109	1.704168
-0.191711	-0.622207	0.824581
-0.191596	0.622314	0.824713
0.000000	0.000000	1.197141
0.000000	0.000000	-1.197141
0.374859	0.000022	-1.877347

H_2O

0.681772	1.829232	-5.664793
0.246723	1.905600	-5.406827
0.200716	1.670037	-4.964689
0.235989	3.604898	-4.850114
-0.237211	1.872643	-5.676976
-1.472777	1.519113	-5.709599
0.246723	1.905600	-5.406827
0.205650	2.336916	-5.059629
0.476386	1.477607	-5.246408
0.377685	2.076703	-5.861435

$^3B_1$ CH_2

-1.844826	0.000000	0.792513
0.000000	0.000000	-0.085869
1.844826	0.000000	0.792513
-0.000015	0.606366	-0.502760
0.000002	-0.606363	-0.502756
-0.801110	-0.000032	0.269010
0.801101	0.000013	0.269022
0.000000	0.000000	-0.085869

$^1A_1$ CH_2

-0.252792	0.000012	-1.315155
0.743957	0.000018	-0.101559
-1.634330	0.000000	0.699766
0.000000	0.000000	-0.606224
0.252775	0.000003	-1.315177
-0.743938	0.000004	-0.101541
0.000000	0.000000	-0.606224
1.634330	0.000000	0.699766

C_3O_2

-1.482635	-0.428461	0.837041
0.000020	-0.417530	-0.856190
0.000000	0.000000	0.072395
-2.284131	0.000000	0.914042
1.482627	-0.428437	0.837088
-3.888211	0.408481	1.818462
2.284131	0.000000	0.914042
-0.000005	0.961547	0.033689
4.116127	-0.406825	1.021140
-4.883063	0.406258	1.262739
-4.116117	-0.406647	1.021012
-4.655068	-0.407591	2.029887

CaH_2

-0.330664	0.274161	-1.033899
-0.034322	-0.076813	-0.509286
-0.322483	0.327825	0.233881
-0.034893	-0.075731	-0.272539
0.142407	0.027286	-0.391018
0.762478	0.142931	-0.397637
0.000433	-3.898100	-1.150698
-0.114994	-0.757404	-0.351835
0.000000	0.000000	-0.391003
-0.073252	0.125261	-0.391987
0.000658	3.613358	-1.085929
-0.000432	3.898100	-1.150698
0.322001	-0.327813	0.234136
-0.142406	-0.027287	-0.391242

MgH_2

-0.132300	0.194855	-0.169679

0.192105	0.135168	0.170514
-0.000003	-0.000003	-3.650725
0.000000	-0.000000	3.612669
0.000000	0.000000	0.000000
0.137869	-0.194977	-0.165061
-0.197672	-0.135037	0.164165
0.132301	-0.194861	0.169640
-0.137867	0.194971	0.165040
-0.000001	-0.000001	-3.610514
0.000000	0.000000	3.658453
-0.192109	-0.135166	-0.170546
0.197670	0.135045	-0.164194
0.000000	0.000000	0.000000

References

Hastings, W. K. (1970). *Biometrika*, 57(1), 97–109.

Liu, D. C. and Nocedal, J. (1989). *Math. Program.* 45, 503–528.

Lüchow, A., Manten, S., Diedrich, C., Bande, A., Scott, T. C., Schwarz, A., Berner, R., Petz, R., Sturm, A., Hermsen, M., Mood, K. H., Schulte, C., Reuter, L., Heuer, M. A. and Ludovicy, J. (2021). Amolqc (v7.1.0). zenodo https://doi.org/10.5281/zenodo.4562745 (2021).

Lüchow, A., Sturm, A., Schulte, C. and Haghighi Mood, K. (2015). *J. Chem. Phys.* 142(8), 084111.

Martín Pendás, A. and Francisco, E. (2023). Promolden. a qtaim/iqa code (available from the authors upon request).

Metropolis, N., Rosenbluth, A. W., Rosenbluth, M. N., Teller, A. H. and Teller, E. (1953). *J. Chem. Phys.* 21(6), 1087–1092.

Pritchard, B. P., Altarawy, D., Didier, B., Gibson, T. D. and Windus, T. L. (2019). *J. Chem. Inf. Model.* 59(11), 4814–4820.

Schmidt, K. E. and Moskowitz, J. W. (1990). *The Journal of Chemical Physics*, 93(6), 4172–4178.

Schmidt, M. W., Baldridge, K. K., Boatz, J. A., Elbert, S. T., Gordon, M. S., Jensen, J. H., Koseki, S., Matsunaga, N., Nguyen, K. A., Su, S. J., Windus, T. L., Dupuis, M. and Montgomery, J. A. (1993). *J. Comput. Chem.* 14, 1347–1363.
